# Graph Properties of Mass-Difference Networks for Profiling and Discrimination in Untargeted Metabolomics

**DOI:** 10.3389/fmolb.2022.917911

**Published:** 2022-07-22

**Authors:** Francisco Traquete, João Luz, Carlos Cordeiro, Marta Sousa Silva, António E. N. Ferreira

**Affiliations:** Laboratório de FT-ICR e Espectrometria de Massa Estrutural, MARE-Marine and Environmental Sciences Centre, Faculdade de Ciências, Universidade de Lisboa, Lisboa, Portugal

**Keywords:** untargeted metabolomics, metabolomics data analysis, mass-difference networks, Fourier transform mass spectrometry, graph properties

## Abstract

Untargeted metabolomics seeks to identify and quantify most metabolites in a biological system. In general, metabolomics results are represented by numerical matrices containing data that represent the intensities of the detected variables. These matrices are subsequently analyzed by methods that seek to extract significant biological information from the data. In mass spectrometry-based metabolomics, if mass is detected with sufficient accuracy, below 1 ppm, it is possible to derive mass-difference networks, which have spectral features as nodes and chemical changes as edges. These networks have previously been used as means to assist formula annotation and to rank the importance of chemical transformations. In this work, we propose a novel role for such networks in untargeted metabolomics data analysis: we demonstrate that their properties as graphs can also be used as signatures for metabolic profiling and class discrimination. For several benchmark examples, we computed six graph properties and we found that the degree profile was consistently the property that allowed for the best performance of several clustering and classification methods, reaching levels that are competitive with the performance using intensity data matrices and traditional pretreatment procedures. Furthermore, we propose two new metrics for the ranking of chemical transformations derived from network properties, which can be applied to sample comparison or clustering. These metrics illustrate how the graph properties of mass-difference networks can highlight the aspects of the information contained in data that are complementary to the information extracted from intensity-based data analysis.

## Introduction

The analysis of the metabolome’s complexity requires analytical methods with high resolution and sensitivity. Mass spectrometry (MS) is a common technique used in metabolomics that allows thousands of metabolites to be detected in a single run. MS-based metabolomics data require several steps of preprocessing that may comprise normalization, transformations, and scaling. These steps aim to highlight the biologically important information in the signal intensities within the data while reducing the impact of undesired variation (van den Berg et al., 2006). For instance, normalization has the goal to eliminate sample variation due to different sources of bias such as sample dilution, sample handling and storage, and heterogeneity of biological tissues analyzed (Katajamaa and Oresic, 2007; Karaman, 2017; Cuevas-Delgado et al., 2020). The result of preprocessing is a two-dimensional numerical matrix, with the features representing retention time and *m/z* values, formulas, or identified compounds in one dimension and the samples in the other dimension. The number of features is usually much higher than the number of samples, and consequently, many are highly correlated, making the subsequent statistical analysis challenging (Vinaixa et al., 2012; Gromski et al., 2015). Furthermore, the metabolome is inherently diverse, consisting of thousands of small molecules involved in complex processes and spatial and temporal organization (Schmitt-Kopplin et al., 2019). Even if quality control measures are taken and metabolism is quenched prior to extraction, reducing the variation observed in metabolite abundances, the metabolome is highly dynamic, with metabolites consistently changing and transforming into each other (Roessner and Hansen, 2007), depending on both internal and environmental conditions (Johnson and Gonzalez, 2012; Worley and Powers, 2013).

In general, untargeted metabolomics’ data analysis focuses on two-dimensional numerical data matrices derived from intensity data to obtain a chemical global characterization of a biological system (Roberts et al., 2012; Bartel et al., 2013) or more often to seek biological class characterization and discrimination while highlighting important biological variables. Nevertheless, metabolomics data can lead to other representations that highlight other types of information content in the data.

**FIGURE 1:**
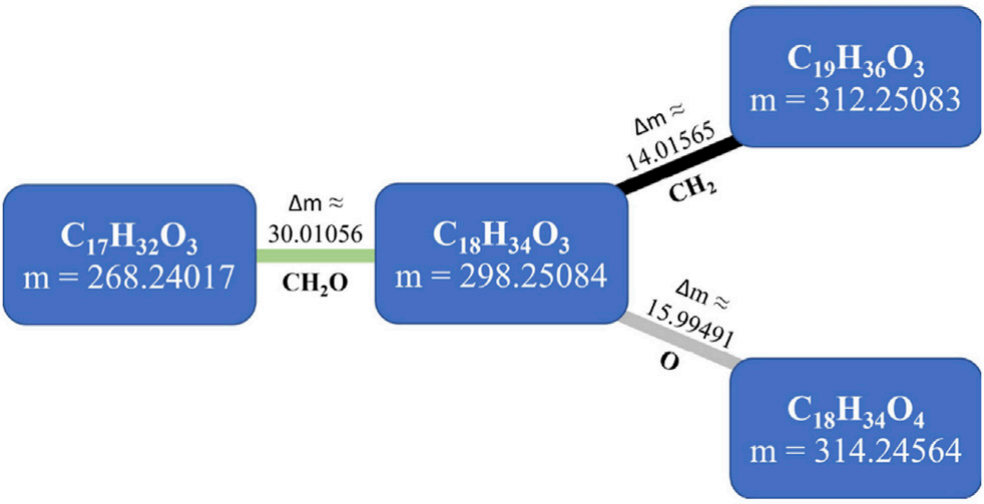
The concept of mass-difference networks (MDiNs). In this four-node example, neutral mass values (in Da) obtained from a mass spectrometry analysis are represented as nodes, connected with mass differences associated with particular mass-difference-based building blocks (MDBs). Δm, mass difference (in Da).

Using extreme mass accuracy (below 1 ppm), together with the ability to resolve isotope ratios, an elemental composition formula can often be assigned to the detected mass features based on possible formulas computed from specific rules for heuristic filtering such as the seven golden rules (Kind and Fiehn, 2007). These formulas can be used to infer metabolite relationships and networks because metabolites can be interconverted through known chemical reactions associated with defined mass differences (Breitling et al., 2006; Moritz et al., 2017). This is the concept behind mass-difference networks (MDiNs), originally developed by Breitling and coworkers (Breitling et al., 2006). These networks use mass values as the nodes of a network. Then, a list of defined mass differences, each representing the occurrence of a chemical transformation, is used to establish edges in the network (Figure 1) (Tziotis et al., 2011; Schmitt-Kopplin et al., 2019). These mass differences have been called mass-difference-based building blocks (MDBs) (Moritz et al., 2017). For example, a methylation (absolute change of CH_2_ after substitution of –H, hydrogen atom, by a –CH_3_ group) would be represented by a mass-difference of 14.01565 Da (Breitling et al., 2006; Tziotis et al., 2011). MDBs can be chosen *a priori* by considering a predefined set of common chemical or biochemical reactions or by the most common mass differences found in a mass spectrum, where a specific chemical transformation can be assigned (Kunenkov et al., 2009; Moritz et al., 2017). Ultrahigh-resolution data allow the construction of nonrandom and informative *ab initio* networks akin to metabolic networks (Breitling et al., 2006) to represent the system’s chemical diversity. MDBs are only based on mass differences, and when compared to metabolic networks, they can consider possible spontaneous nonenzymatic reactions. Moreover, they are not influenced or skewed by previous knowledge on metabolic pathways that are still very incomplete in many less studied biological systems with largely unknown metabolomes (Breitling et al., 2006; Moritz et al., 2017), such as plant metabolomes (Lee et al., 2020).

MDiNs are one of the major types of experimental networks used for metabolomics data analysis (Amara et al., 2022) and have been primarily used for molecular formula assignment and annotation (Tziotis et al., 2011; Moritz et al., 2017) or the depiction of a system’s chemical diversity (Tziotis et al., 2011). Because each edge has a corresponding absolute change of metabolite formulas (addition or subtraction), the formula assignment of an entire network component can be made from a single reliably annotated node through formula propagation (Breitling et al., 2006; Tziotis et al., 2011; Moritz et al., 2017; Amara et al., 2022). As a few examples, MDiNs have been used in this way in studies on mouse cecal samples (Walker et al., 2014), yeast (Liu et al., 2016), and mouse adipose tissue and human blood plasma (Laber et al., 2021), among others (Moritz et al., 2015; Willkommen et al., 2018). Furthermore, other methods similar to MDiNs that use mass differences for formula annotation have been developed (Weber and Viant, 2010; Morreel et al., 2014).

Despite being originally aimed at high-resolution metabolomics data obtained in instruments such as FT-ICR or Orbitraps, MDiN-based molecular formula assignment was used for lower resolution LC-MS data if the right steps were taken, such as starting formula propagation from high-confidence annotated metabolites (Forcisi et al., 2015).

Another recent application of MDiNs has been the characterization of biological groups based on their different prevalence of MDBs/chemical transformations *via* a mass-difference enrichment analysis to test MDB over- or under-representation in the edges connected to a set of nodes of interest compared to their overall prevalence in the MDiN (Moritz et al., 2017, 2019). The potential of this methodology has been highlighted in the characterization of different biological systems (Moritz et al., 2015, 2017; Clancy et al., 2018; Kaling et al., 2018; Laber et al., 2021). An in-depth review of MDiNs and all types of networks used in metabolomics has recently been published (Amara et al., 2022).

In this work, we apply the concept of MDiNs to characterize biological systems by using them as the starting point for class profiling and discrimination, as an alternative to starting from two-dimensional intensity data matrices. For this purpose, we focus on the properties of MDiNs as graphs, building a different MDiN for each sample in a dataset (sample MDiNs or sMDiNs), as the basis for class discrimination and feature importance assignment. Our fundamental assumption is that the set of detected metabolites and their possible chemical connections are a characteristic of each sample in a study. As a consequence, the graph properties of MDiNs must also be a characteristic. sMDiNs are built based on the occurrence of spectral features, disregarding intensity information prominently used in common data analysis workflows, taking advantage that the former is less prone to variation. Graph analysis methods fulfill the same role as the data pretreatments applied to two-dimensional numerical data matrices. However, instead of focusing on maximizing the contribution of significant information in the signal intensities, these methods can focus on different aspects of the network such as node centrality, network topology, and edge properties, and thus, they may provide different and complementary results and insights. Furthermore, feature importance assignment from sMDiNs can be based on this network characterization with the potential to be very versatile.

To demonstrate the use of sMDiNs for class characterization and discrimination, we performed an empirical comparison of the performance of unsupervised and supervised analysis methods applied to profiles associated with the graph metrics of each sMDiN. The performance of these methods is compared with the performance obtained with the same datasets by applying a traditional intensity-based pretreatment, representing the typical metabolomics data analysis preprocessing pipelines. We conclude that sMDiNs keep essential information for class discrimination and do not degrade the performance of statistical methods, especially when node centrality analysis was applied, for both low and high sample number per class, and two-class and multiclass datasets. Furthermore, we show how further feature importance assignment, such as weighted MDB impact, can provide extra insights regarding our data and can complement the discrimination analysis. On the basis of this work, we believe that network analysis methods have the potential to extract complementary meaningful information from ultrahigh-resolution metabolomics datasets.

## Materials and Methods

### Datasets

Five data matrices were built from experimental untargeted metabolomics data with different numbers of features and missing-value abundance. These matrices (datasets) were based on three ultrahigh-resolution metabolomics studies: two obtained from Fourier transform ion-cyclotron resonance mass spectrometry (FT-ICR-MS) instruments, with different mass accuracy, resolution, and sensitivity, and one obtained from an Orbitrap instrument after hydrophilic interaction chromatography (HILIC).

The “grapevine dataset” (GD) was built from published data related to a study of the metabolome differences observed in *Vitis* varieties susceptible or resistant to oomycete/fungal infections (Maia et al., 2020a) and openly available at a public repository (Maia et al., 2020b). Metabolomics data from three biological replicates of 11 different grapevine genotypes were collected by direct infusion in a 7T-Apex FT-ICR-MS, operating in negative electrospray ionization mode (Maia et al., 2020a). Data alignment was performed using the metabolinks Python package (Ferreira and Traquete, 2021), as previously described (Traquete et al., 2021). From this matrix, two datasets were created by different filters: dataset *GDg2* by only retaining features that occur globally at least twice in the 33 samples and dataset *GDc2* by only retaining features that occur at least twice in at least one *Vitis* variety (class) were retained. Furthermore, the *GD types* matrix was generated for the assessment of the performance of supervised methods in two-class problems by changing the dataset class labels in *GDc2* according to whether grapevine is from *Vitis vinifera* or wild *Vitis* (non-*vinifera*) species.

The “yeast dataset” (*YD*) was built from data related to a study to discriminate single-gene deletion yeast mutants (mostly affecting genes coding enzymes involved in methylglyoxal metabolism) with FT-ICR-MS (Luz, 2021; Sousa Silva et al., 2021) and openly available at a public repository (Luz et al., 2021). In brief, metabolomics data from three biological replicates of five different yeast isogenic strains were collected by direct infusion in a 7T Solarix XR FT-ICR-MS (Luz, 2021; Sousa Silva et al., 2021) – wild type (WT) and ΔGLO1, ΔGLO2, ΔGRE3, and ΔENO1. Data alignment was performed with MetaboScape 4.0 software (Brüker Daltonics, Germany), following the procedure previously described (Traquete et al., 2021). From the resulting “bucket table” (representing the neutral masses of the detected metabolites), formulas were assigned with annotation from the Human Metabolome (HMDB) (Wishart et al., 2018) and Yeast Metabolome (YMDB) (Ramirez-Gaona et al., 2017) databases and then with MetaboScape’s SmartFormula algorithm (considering the following parameters: *m/z* tolerance narrow 0.1 and wide 1.0 ppm and mSigma narrow 10 and wide 100; elements considered: C, H, N, O, S, and P with the “Auto Upper Formula” option with at least one carbon and one hydrogen; elemental ratios allowed: H/C, 0.2–3.1; O/C, 0.0–1.5; N/C, 0.0–1.3; S/C, 0.0–0.8; P/C, 0.0–0.3; and P/O, 0.0–0.34). The Senior and Lewis MetaboScape filter and the heuristic element count probability were applied. When the same formula was assigned to two different bucket labels, these were joined together. Data matrix *YD* was constructed by retaining features that occurred in at least two samples.

The “human dataset” (*HD*) was constructed from data obtained from a study of the preoperative metabolic signatures related with prostate cancer recurrence or remission after a radical prostatectomy, obtained in an Orbitrap instrument (Clendinen et al., 2019) and publicly available at the NIH Common Fund’s National Metabolomics Data Repository website, the Metabolomics Workbench, with project ID PR000724 (study ID: ST001082). Metabolomics data from 80 patients’ blood serum were collected before radical prostatectomy and analyzed using HILIC coupled to a Thermo Q Exactive HF hybrid Orbitrap operating in positive electrospray ionization (ESI+) mode (Clendinen et al., 2019). Data alignment was performed with the Progenesis QI software package (Nonlinear Dynamics, Waters Corp., Milford, MA, United States) (Clendinen et al., 2019). Data consist of 135 MS spectra samples from patients in prostate cancer remission and 114 samples from patients with prostate cancer recurrence (“no recurrence” versus “recurrence”). From these samples, an average of five blank samples was subtracted, with negative values being coded as missing values. Features that appeared in only one sample were excluded. Multiple features with the same *m/z* value (but different retention times) were treated as having the same mass value.

An overview of the five datasets (*GDg2*, *GDc2*, *GD types*, *YD*, and *HD*) characteristics is described in Table 1. A preliminary assessment of the extent of class proximity in the datasets and therefore the degree of difficulty for the statistical downstream analysis methods is presented as principal component analysis score plots in Supplementary Figure S1.

**TABLE 1:** General characteristics of the different benchmark datasets.

Dataset	Samples	Features	Classes	Features/sample (range)	Samples/class
*GDg2*	33	3,629	11	658 (367–1002)	3
*GDc2*	33	3,026	11	547 (338–919)	3
*GD types*	33	3,026	2	547 (338–919)	15 *Vitis vinifera*, 18 wild *Vitis*
*YD*	15	1,893	5	646 (559–705)	3
*HD*	249	12,869	2	7936 (7057–8475)	114 “Recurrence,” 135 “no Recurrence”

### Intensity-Based Data Pretreatment

As a benchmark comparison for the different network methods applied to sample MDiNs, an intensity-based data pretreatment (IDT) was applied independently to all datasets. Missing values were imputed either by replacing them with one-fifth of the minimum of nonmissing values in each sample (1/5 min) or by random forest (RF) missing-value imputation (Stekhoven and Bühlmann, 2012). The number of trees in RF was set to 50, and the number of similar features used for prediction was 100. These imputation methods were chosen because RF imputation has been found to outperform most other imputation strategies in most different cases of prevalence and types of missing values for metabolomics data, except when values are mostly missed not at random, which is the situation where features are absent or below the detection limit in a sample. In the latter case, limit of detection-related procedures, such as 1/5 min, can outperform RF imputation (Wei et al., 2018; Kokla et al., 2019). After missing-value imputation, the *GD* and *YD* datasets were normalized by the reference feature leucine enkephalin (for ESI-data, *m/z* 554.262022) whereas the *HD* dataset was normalized by probabilistic quotient normalization (Dieterle et al., 2006) using the mean of all samples as the reference. Data were then transformed by the generalized logarithmic transformation and Pareto scaled.

In all of the results of this study obtained with the IDT-treated datasets, we report only the best value of the downstream performance methods obtained with the IDT using either one of the two of the missing-value imputation procedures.

### Mass-Difference Network Generation

MDiNs were generated for each benchmark dataset using the MetaNetter 2.0 plugin (Burgess et al., 2017) of Cytoscape 3.8.1 (Shannon et al., 2003). Each network was built using all neutral masses of the mass peaks of each aligned dataset as nodes. Edges are defined as the mass-difference between two mass values caused by a chemical transformation, MDBs. The list of 15 MDBs used to build each network was defined *a priori*, and it is indicated in Table 2. Edges were established in each benchmark dataset by MetaNetter 2.0 with an accepted deviation of 1 ppm. The MDBs were chosen to represent some of the most common and ubiquitous small reactions in biological systems (both enzymatic and nonenzymatic). Representative MDBs were searched using the BRENDA enzyme database (Jeske et al., 2019). The choice of the set of MDBs is crucial because the structure of the network directly depends on these and can be highly specific to the biological problem presented. As this work focuses on the general viability of the methodology, only a very restricted set of MDBs expected to occur in almost every biological system was used. To this end, only MDBs representing changes of less than 80 Da in a metabolite, while still maintaining metabolite neutrality, were considered. For example, phosphorylation is represented by the MDB PO_3_H and corresponds to the addition of a –PO_3_^2−^ group plus 2 H^+^ (to maintain neutrality) while replacing an H atom. All common elements in metabolites (C, H, O, N, S, and P) are represented in at least one of the selected MDBs. For the *YD* dataset, two extra MDBs were considered, representing either direct or indirect reaction mechanisms associated with glycation, leading to carboxymethylation and carboxyethylation (Table 2) (Requena et al., 1997; Shoji et al., 2010). The node and edge number, percentage of connected nodes, and size, radius, and diameter of the biggest component were calculated for each dataset’s MDiN.

**TABLE 2:** List of MDBs used to build the MDiNs.

Elemental transformations (MDBs)	Δ Mass (Da)	Reaction type examples
O(–NH)	0.984016	Deamination
NH_3_ (–O)	1.031634	Transamination
H_2_	2.015650	Hydrogenation/Dehydrogenation
CH_2_	14.015650	Methylation
O	15.994915	Oxygenation/Hydroxylation
H_2_O	18.010565	Condensation/Dehydration/Cyclization
NCH	27.010899	Transfer of a formidoyl group
CO	27.994915	Formylation
CHOH	29.002740	Hydroxymethylation
S	31.972071	Transfer of a –SH group
C_2_H_2_O	42.010565	Acetylation
CONH	43.005814	Transfer of a carbamoyl group
CO_2_	43.989829	Carboxylation/Decarboxylation
CHCOOH*	58.005479	Carboxymethylation
CCH_3_COOH*	72.021129	Carboxyethylation
SO_3_	79.956815	Sulfation
PO_3_H	79.966331	Phosphorylation

The mass variation (Δ Mass, in Da) corresponds to specific changes in the elemental composition of a metabolite and is associated with certain types of reactions. MDBs marked with * were exclusively used for the YD dataset.

Sample MDiNs for each sample in each benchmark dataset were built by subgraphing the dataset MDiN with only the nodes characteristic of that sample. This is equivalent to building the sMDiNs for each sample from scratch because establishing an MDB edge only depends on the masses of the two nodes linked and no other edge or node. Nodes with degree 0 (establishing no connections and, therefore, uninformative) were excluded.

### Graph Metrics for Sample Mass-Difference Networks

The generated sample MDiNs were analyzed using six different metrics. For each combination of dataset and metric, a data matrix could be derived for profiling each sample with that particular graph metric. These data matrices are amenable to be analyzed by conventional statistical methods in a fashion identical to the methods employed with intensity-based data.

Three node centrality metrics were used:

• Degree: the number of connections with neighboring nodes.
• Betweenness centrality.

$$\mathrm{Betweenness centrality}\left(\mathrm{node v}\right)=\sum_{\mathrm{s,t \varepsilon V}}\frac{\sigma \left(\mathrm{s, t|v}\right)}{\sigma \left(\mathrm{s, t}\right)}$$ (1)

• Closeness centrality.

$$\mathrm{Closeness centrality}\left(\mathrm{node v}\right)=\frac{\mathrm{n-1}}{\mathrm{N-1}}\times \frac{\mathrm{n-1}}{\sum_{\mathrm{u=1}}^{\mathrm{n-1}}d\left(\mathrm{u,v}\right)}\;$$ (2)

In Eqs. 1 and 2, V is the set of all nodes, σ (s, t) is the number of shortest paths between node s and t, σ (s, t|v) is the number of those paths that pass through v (with v different from s or t), n is the number of nodes that can reach node v, N is the number of nodes in the graph, and d (u, v) is the distance of the shortest path between node v and node u (Hagberg et al., 2008).

The resulting data matrices maintained the *m/z* peaks (network nodes) as features, with their values in each sample being the respective value of the centrality metric in the corresponding sMDiN.

Two metrics focused on quantifying the influence of each MDB in the construction of the sMDiNs were also employed: the MDB impact (MDBI) and weighted MDB impact (WMDBI). These metrics were inspired by a similar concept introduced by Moritz and coworkers to identify metabolome differences and over-representation of reactions between two gray poplar genotypes (Moritz et al., 2017). MDBI is the fraction of edges attributed to each MDB of the total number of edges. Using these fractions as features in each sample greatly reduces the number of features from 1000s to 15 to describe the sMDiNs. In weighted MDBI, each edge is weighted by the sum of the importance of the nodes it links. The importance of these nodes was estimated by their gini importance in the RF models based on the degree profiles, described earlier.

The last analysis method was the graphlet correlation distance, which includes the 11 nonredundant orbits of up to four-node graphlets (GCD-11) to express the network topology (Yaveroğlu et al., 2014). Graphlets are small and nonisomorphic subgraphs of a network. Each of these subgraphs can have multiple automorphic orbits if the nodes in the graphlet are not in the same relative position (Milenković and Pržulj, 2008). In this method, a matrix for an sMDiN is built by counting the number of times that each node of the network is in each of the 11 orbits. The Spearman correlation between the columns (11 orbits) of the matrix makes an 11 × 11 symmetric matrix called the graphlet correlation matrix signature of the network topology (Yaveroğlu et al., 2014; Tantardini et al., 2019). Here, the method was adapted, and a data matrix was built using 60 correlations between different orbits as the features for each sample of the benchmark datasets. Thus, the number of features is greatly reduced from the original data matrix.

### Clustering Methods (Unsupervised Analysis)

The discriminatory power based on the differences between sMDiNs was assessed by comparing the effects of the performance of clustering and classification methods on the data matrices obtained from the network analysis methods on sMDiNs and on the intensity-based pretreated data. Two typical types of unsupervised methods were applied: agglomerative hierarchical clustering (HCA) and K-means clustering. Clustering performance was assessed by the methods’ ability in correctly clustering samples belonging to the same class. These classes, as defined by wild *Vitis* versus *Vitis vinifera* plants in *GD types*, by samples from patients with cancer recurrence versus remission in *HD*, and by the replicates in the remaining datasets, are the “ground truth” of correct clusters and allow the use of ground-truth-related metrics of clustering performance.

Agglomerative hierarchical clustering analysis with unweighted pair group method with arithmetic mean (UPGMA) linkage method and Euclidean distance was performed on each IDT-treated dataset and the matrices obtained from each sMDiN network analysis metric. Three metrics were used to express clustering performance. The “correct clustering” percentage is defined as the percentage of the classes whose samples all clustered together before clustering with other samples or already-formed clusters in the agglomerative procedure. The “discrimination distance” is defined based on the average of “class discrimination distance.” For each class, if it is “correctly clustered” (defined as in the metric before), the discrimination distance is the distance between the node that includes all samples of the class and the next closest node (including those samples) in the agglomerative procedure, normalized by the maximum distance of any pair of nodes in the clustering; if not, it is 0. The “correct first cluster” percentage is defined as the percentage of samples whose first time that they clustered was only with sample(s) from its own class.

K-means clustering analysis was applied using the Euclidean distance. The cluster number was equal to the total number of classes in each dataset (11 in *GD*, 5 in *YD*, and 2 in *GD types* and *HD*). Owing to the randomness of the initial centroid assignments (Andreopoulos et al., 2009), the algorithm was iterated 20 times and the result with the least inertia was retained. Clustering performance was also assessed by three metrics: discrimination distance and correct clustering percentage (class-based metrics) were computed by considering the distances between cluster centroids. For K-means, a “correct clustering” is defined as the cluster containing all and only the samples of a single class (total homogeneity and completeness). As a stricter condition imposed in HCA for correct clustering, a lower correct clustering percentage is expected. The third metric focused on the samples was the Rand Index, defined by the proportion of sample pairs that are correctly clustered or correctly not clustered, adjusted for the expected proportion with samples randomly clustered. For the two-class datasets (*GD types* and *HD*) that have a larger number of samples per class, only the correct first cluster percentage and Rand Index were considered. This choice is justified by the higher likelihood that clustering percentage and discrimination distance will be close to zero because a single sample behaving as an outlier can make all other samples of a class being considered not correctly clustered.

### Classifier Methods (Supervised Analysis)

Random Forest and projection in latent structures–discriminant analysis (PLS-DA) were chosen as classifiers for the comparison between the IDT and several sMDiN graph properties. The classes for prediction were defined as identical to the ground-truth groups of the clustering analysis. The performance of the classifiers was assessed by their predictive accuracy. *GD types* accuracy was evaluated by fivefold stratified cross-validation. The *HD* dataset was evaluated on a test set resulting from a 70%/30% random train/test split due to its large sample size. The other datasets, considering their classification targets, have a low number of samples per class, and consequently, accuracy was evaluated by internal stratified threefold cross-validation (Lee et al., 2018).

For each classifier, 20 iterations of different cross-validation folds were performed. To avoid data leakage, for models based on the IDT, for each cross-validation fold and each iteration, the train and test data were treated independently by the same transformation pipeline. For the treatment that included RF missing-value imputation, the training and test sets were filtered to only include features appearing in at least two samples of the training set. Features of the test set occurring in only one sample were imputed by one-fifth of the minimum, instead of RF imputation. Features that did not appear in the test set were added to this one with the minimum value appearing in the training set. In the case of WMDBI, as it requires previous node degree importance assignment, these were computed from RF models using only the training set of that iteration/fold combination. The remaining sMDiNs metrics are a characteristic of each sMDiN, so these procedures to avoid data leakage do not need to be applied. Except for the *HD* dataset, the results presented are the average results of the 20 iterations.

After the optimization of the number of trees to 100, RF classifiers were built with the scikit-learn RandomForestClassifier object constructor, leaving other parameters as their default values. The gini importance of each feature was calculated for each model (Louppe et al., 2013). For the two-class classification problems (*GD types* and *HD*), receiver operating characteristic (ROC) curves were computed for the IDT transformed data and for every sMDiN analysis with fivefold stratified cross-validation.

PLS-DA classifiers were built using the PLS2–NIPALS algorithm implemented in PLSRegression of scikit-learn (Pedregosa et al., 2011). The numbers of components chosen were 10 for the *GD* and *HD* datasets and 6 for the *YD* and *GD types* datasets after optimization based on maximizing Q^2^, that is, minimizing the predictive residual sum of squares (computed from internal stratified cross-validation). For the matrices obtained from MDBI and WMDBI sMDiN analysis, four and six components were used, respectively. The data matrices obtained from the network analysis of sMDiNs were auto-scaled during the development of the PLS-DA models, whereas IDT-treated data were already scaled. For multiclass problems, one-hot encoding was used to encode class membership and the decision rule for class prediction was employed to assign to samples the class corresponding to the maximum value in ypred of the PLS output. For two-class problems, class membership was coded as 0 or 1, with 0.5 threshold for decision. For both classifier methods, permutation tests (with 500 iterations each) were performed to further assess model significance.

### Implementation

Data pretreatments, analysis of the sMDiNs, clustering methods, and classifiers were implemented in Python language version 3.9.7 using the following packages: pandas version 1.4.1 (McKinney, 2010), numpy version 1.20.3 (Harris et al., 2020), scikit-learn version 1.0.1 (Pedregosa et al., 2011), networkx version 2.6.3 (Hagberg et al., 2008), scipy version 1.7.3 (Virtanen et al., 2020), matplotlib version 3.5.0 (Hunter, 2007), seaborn version 0.11.2 (Waskom et al., 2020), and metabolinks version 0.71 (Ferreira and Traquete, 2021). The code that supports this study is available at the repository https://github.com/ftraquete/paper_sMDiN.

## Results

### Mass-Difference Network Construction and Analysis

One MDiN was built from each benchmark dataset using the MDBs indicated in Table 2. sMDiNs were derived by subgraphing the dataset MDiNs with only the features appearing in each sample. Sample MDiNs were analyzed by six different network analysis methods, and for comparison, the datasets were also treated by the intensity-based data pretreatment (IDT).

**FIGURE 2:**
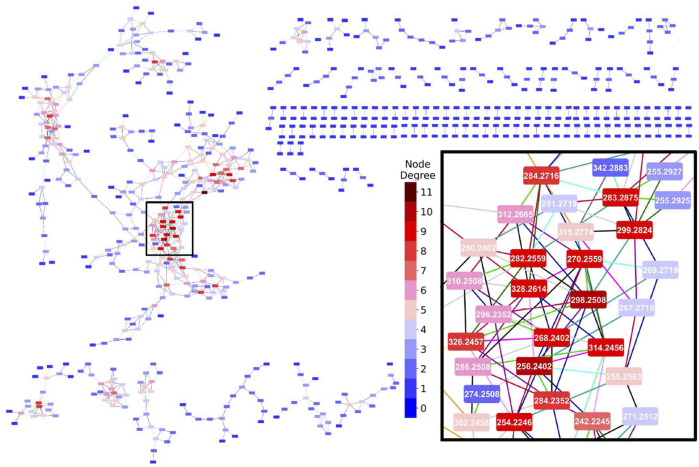
Mass-difference network built from the *YD* dataset. The inset is a close-up of the selected rectangle in the populated area of the largest network component. Edge colors represent each MDB: (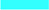) – O(–NH), (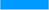) – NH_3_(–O), (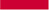) – H_2_, (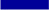) – CH_2_, (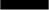) – O, (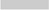) – H_2_O, (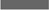) – NCH, (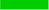) – CO, (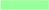) – CHOH, (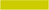) – S, (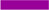) – CH_2_O, (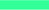) – CONH, (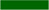) – CO_2_, (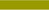) – SO_3_, (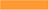) – PO_3_H, (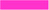) – CHCOOH, and (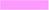) – CCH_3_COOH. Node background colors represent the node degree. Network representations were made with Cytoscape 3.8.1 (Shannon et al., 2003).

The characteristics of the MDiNs derived from the five benchmark datasets are shown in Table 3 and a full MDiN built from the *YD* dataset, with a close-up on its most populated area, is depicted in Figure 2. A representation of the largest components of the MDiNs of the remaining datasets is shown in Supplementary Figure S2.

**TABLE 3:** General characteristics of the mass-difference networks built from the different datasets.

Dataset MDiNs	Nodes	Edges	Size of largest component	Connected nodes (%)	Diameter	Radius
*GDg2*	3,629	1,005	183	32.43	27	14
*GDc2*	3,026	718	145	29.31	31	16
*GD types*	3,026	718	145	35.60	31	16
*YD*	1,893	914	291	37.35	30	15
*HD*	12,867	31,008	7,631	74.94	63	32

Diameter and radius are calculated for the largest network component.

The MDiNs had similar overall characteristics. Only approximately one-third to half of the nodes established connections. The networks were, therefore, somewhat sparse, although *HD* sMDiNs were denser because of the high number of features (Table 1). Unconnected nodes were uninformative for posterior network analysis, and they were excluded. A main and larger component was usually present. This component had a center with a zone(s) composed of nodes with a higher degree, which acted as main hubs, as shown in Figure 2. The high diameter and radius (half of the diameter) showed that the networks were highly spread out from those hubs. The network topology with a low number of nodes with high degree and a high number of nodes with low degree (where the degree distribution approximates a power law) was expected because this was a characteristic of many different biological systems, including metabolic networks (Barabási and Oltvai, 2004). sMDiNs had this same overall topology on a smaller scale. The MDBs that contributed the most to the edges in the MDiNs of all datasets were CH_2_ (methylation), H_2_ (hydrogenation), and O (oxygenation and hydroxylation) (Supplementary Table S1), which are, as expected, some of the most prevalent chemical reactions in biological systems.

To assess the viability of using sMDiNs as the information basis for class discrimination, the performances of two clustering methods (HCA and K-means) and two classifiers (RF and PLS-DA) trained on data obtained from the application of different network analysis and with data obtained with IDT were compared. The benchmark datasets included three examples of low sample number per class and more than two classes (*GDg2*, *GDc2*, and *YD*) and two-class problems with a high number of samples per class (*GD types* and *HD*) and also obtained in different high-resolution instruments in order to empirically test the use of sMDiNs against an array of different types of metabolomics data, easier or harder to discriminate.

### Unsupervised Methods—Hierarchical and K-Means Clustering

**FIGURE 3:**
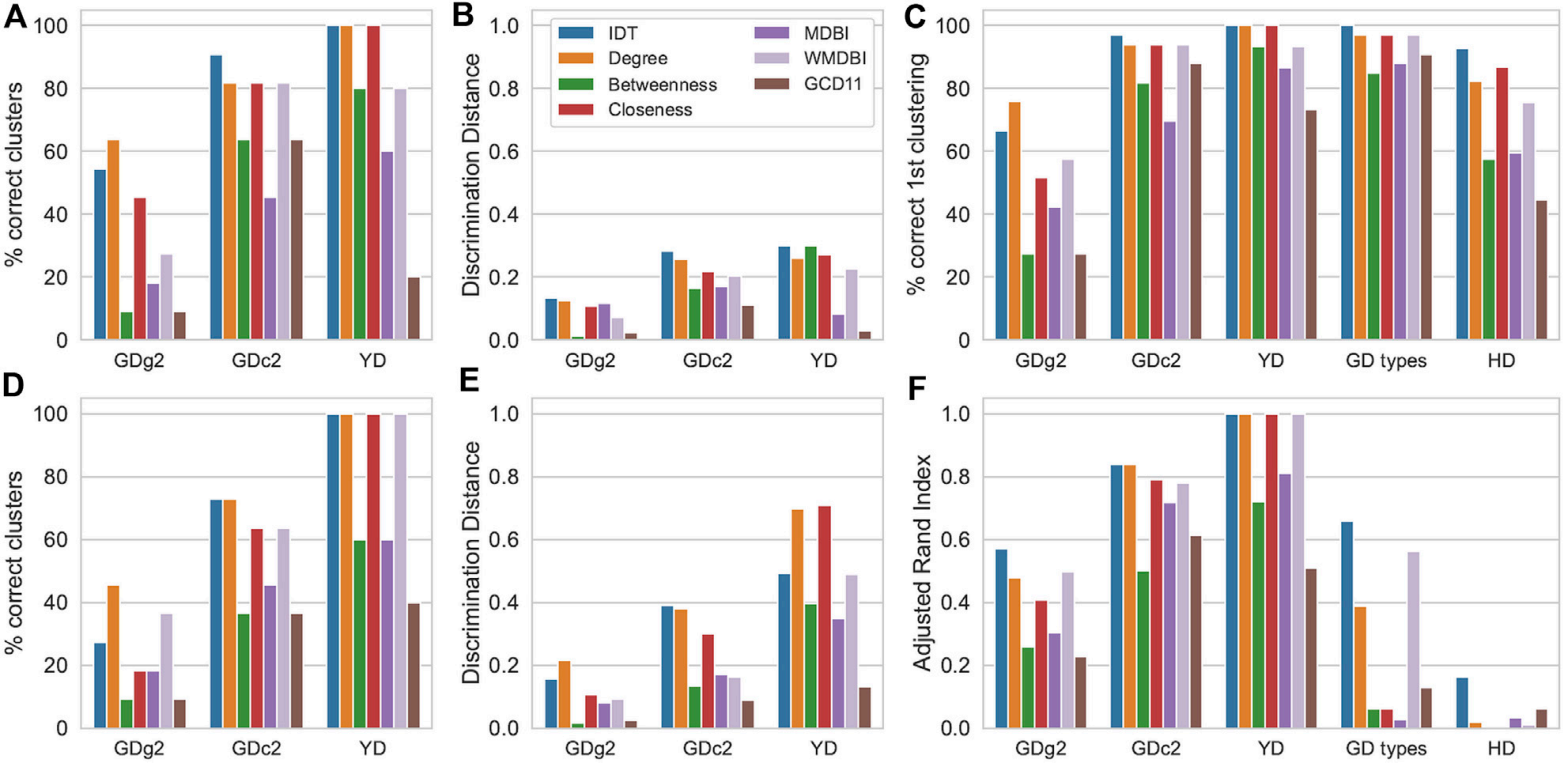
Effect of IDT and sMDiN graph property analysis on clustering performance. **(A)** Correct clustering in HCA; **(B)** discrimination distance in HCA; **(C)** correct first clustering in HCA; **(D)** correct clustering in K-means clustering; **(E)** discrimination distance in K-means clustering; **(F)** adjusted Rand Index in K-means clustering. Methods are as follows: intensity-based data pretreatment (IDT); network analysis: degree analysis (degree), betweenness centrality analysis (betweenness), closeness centrality analysis (closeness), MDB impact (MDBI), weighted MDB impact (WMDBI), and GCD-11 topology analysis (GCD11).

The results of clustering are shown in Figure 3. Performance was assessed by the ability to cluster the samples of the same class together (defining ground-truth clusters), that is, by the correct clustering percentage, discrimination distance, correct first cluster percentage (for HCA only), and the Rand Index adjusted for randomness (for K-means only). As an HCA example, resulting dendrograms from IDT-treated data and degree, MDBI and weighted MDBI sMDiN network analyses of the *GDc2* dataset are shown in Supplementary Figure S3.

The clustering results applied to the data from the degree analysis tended to outperform all other graph metrics employed. Degree profiles also often performed very similar to IDT. Considering correct clustering (Figures 3A, D), both clustering methods often achieved the highest values with data generated from the degree analysis, except for *GDc2* for HCA, where it trailed behind IDT. For *YD*, the perfect clustering obtained with IDT was also achieved with the degree analysis. Discrimination distance (Figures 3B, E), a measure to represent the robustness of clustering discrimination performance to outliers (Traquete et al., 2021), followed the same trend with high proximity between IDT and degree analysis of sMDiNs, with only a considerable lead of the degree analysis on the K-means clustering analysis of *YD*. The results of these three datasets were qualitatively the same for the correct first clustering percentage for HCA and for the Rand Index for K-means that offer finer granularity (Figures 3C, F).

For HCA, in the case of *GD types* and *HD* datasets, we could observe that the IDT pretreatment slightly outperformed degree and closeness analysis. However, for K-means clustering, the Rand Index plummeted (in comparison to the correct first cluster percentage) for all methods. Thus, despite the better performance of IDT, K-means clustering for these two larger datasets with two groups cannot cluster well the samples into the two expected clusters.

Focusing on the other node-centric network methods, closeness centrality led to results only slightly below or equal to those of degree analysis, slightly outperforming it on occasion, whereas betweenness centrality resulted in considerable worse performance. Considering all node-centric analyses employed, degree stands out as the best and the less computationally expensive to calculate.

Considering global network characteristics, both MDBI and GCD-11 resulted in subpar clustering discrimination in comparison to the degree profiles, although, in general, MDBI leads to better results compared to GCD-11. A reason for this might be the aggressive truncation of features to 15 (or 17 for *YD*) in MDBI and 60 in GCD-11 that might not be enough to discriminate the 30+ samples of *GD* data. Nevertheless, the fact that GCD-11, which focuses on network topology (60 features), performs poorly in comparison to MDBI (15 features) indicates that the overall topology of the sMDiNs might not be sufficiently distinctive for group discrimination. In contrast, WMDBI, a modified MDB impact metric that weights the edges based on the importance of the nodes it links, performed considerably better than MDBI and often similar to degree or closeness. It is worth noticing that the importance of the nodes was estimated by their gini importance in a RF (supervised) model based on the data of the degree analysis. We followed the assumption that the most important nodes from the degree analysis will be those that are more characteristic of the different classes. Thus, the edges of these nodes will also be more important and will then be given a higher weight. Degree profiles led to the best results, showing that they contain significant information for profiling. Clustering based on WMDBI is thus not the result of a purely unsupervised analysis, which might explain the very good performance. Despite the encouraging results with clustering, supervised methods are expected to be more appropriate to assess the relative merits of WMDBI.

The dendrograms for dataset *GDc2* (Supplementary Figure S3) illustrate these trends. Degree analysis and IDT-treated data led to an almost recovery of ground-truth sample groups, which was not observed in the dendrograms obtained from MDBI and weighted MDBI. However, the four dendrograms present similarities at higher level clustering such as the proximity between CS and RL samples, which indicates a higher level structure in the data maintained between the different network methods and IDT. Degree and IDT dendrograms present even more similarities such as the grouping of CAN, RIP, LAB, and SYL samples.

These results suggest that using sMDiNs as a basis for clustering is possible because they retain the important discriminatory information to allow for group discrimination. Furthermore, degree (node-centric) analysis of the sMDiNs sometimes even slightly outperformed the intensity-based pretreatments, being the best of the six graph properties compared here.

### Supervised Statistical Analysis—Random Forest and Projection in Latent Structures Discriminant Analysis Classifiers

**FIGURE 4:**
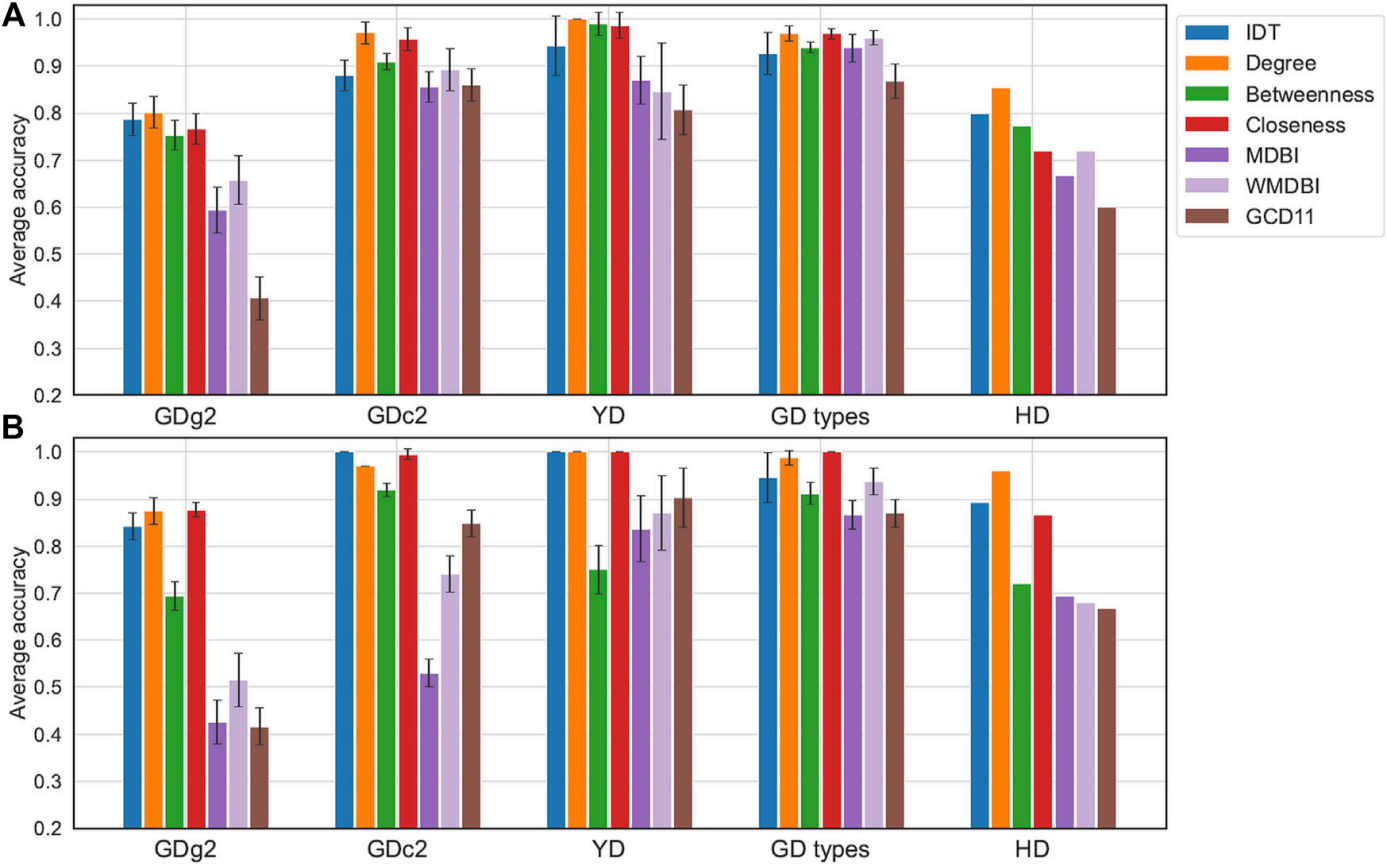
Classification performance of models developed from IDT-treated data or sMDiN graph property methods. **(A)** Performance of random forest (RF) models; **(B)** performance of projection in latent structures–discriminant analysis (PLS-DA). For all datasets except *HD*, accuracy was estimated by 20 iterations of internal three- or fivefold stratified cross-validation, with the error bars representing the accuracy standard deviation. For the *HD* dataset, accuracy was estimated on a test set resulting from a stratified random 70/30% train/test split. Methods are as follows: intensity-based data pretreatment (IDT); network analysis: degree analysis (degree), betweenness centrality analysis (betweenness), closeness centrality analysis (closeness), MDB impact (MDBI), weighted MDB impact (WMDBI), and GCD-11 topology analysis (GCD11).

We compared the predictive performance of different classifiers applied to the benchmark datasets, considering the classes as target labels. *GD types* and *HD* illustrated the use of sMDiNs as a basis for class discrimination with two-class problems, a common scenario in metabolomics data analysis, and the remaining examples were multiclass. Performance was assessed by the model’s predictive accuracy. The classifiers chosen were RF and projection in latent structures–discriminant analysis (PLS-DA) due to their popularity in metabolomics data analysis. These classifiers were applied to data matrices obtained from IDT and the six network analysis methods. Average accuracy results are shown in Figure 4. For the *HD* dataset, owing to its large sample size, results shown are based on the model’s prediction accuracy on a test set (30% of samples).

The RF and PLS-DA classifier performance results were similar, with sometimes PLS-DA models leading to noticeable better accuracies (*GDg2* or *HD*), and followed trends alike those observed in clustering analysis. Classifiers developed for node centrality sMDiN methods performed better than those for global network characteristics (MDBI or GCD-11). RF classifiers from betweenness centrality profiles had comparable performances to the other centrality measures, but the corresponding PLS-DA classifiers show a sharp drop in performance (usually 0.2 to 0.3 lower). Degree profiles are just slightly better than closeness profiles in general, while having sometimes marginally lower accuracies than closeness centrality.

The RF accuracies of IDT-based models are lower than those of PLS-DA, except for the *GD types* dataset. Degree profile RF classifiers outperformed IDT RF classifiers for *GD*, *YD* (achieving perfect prediction), and *HD*. In fact, for *GD types* and *GDc2*, most models based on network metrics outperformed those based on IDT-treated data. For PLS-DA, however, IDT and degree analysis led to more similar accuracies, being nearly identical for most datasets. Interestingly, only the PLS-DA model built from closeness centrality achieved perfect accuracy for *GD types*.

ROC curves computed for RF models of *GD types* and *HD* datasets showed the good performance of the network analysis, with degree slightly outperforming the IDT for *GD types* and IDT having slightly higher area under the curve (AUC) than the degree analysis (Supplementary Figure S4).

RF models built from WMDBI and MDBI outperformed the corresponding PLS-DA models. Among these, WMDBI almost always outperformed MDBI, showing the improved performance that is gained by weighting the edges according to their importance for class discrimination (assessed by the RF model based on the node degree profiles). This also shows the potential of articulating multiple sMDiN metrics that look at different data aspects to improve our findings showcasing the versatility of this analysis. Furthermore, despite underperforming IDT-based and node centrality-based models, the decent performance achieved using WMDBI with only 15 or 17 features revealed that discriminatory information is being compiled into these features. Because sMDiNs have discriminatory information and these results showed that this information content is embedded in WMDBI analysis, we believe that it is viable to use this metric as a means to perform feature importance analysis to rank chemical transformations for the purpose of using their importance profile in sample discrimination.

Permutation tests were made to assess the significance of the accuracy of the models (Supplementary Table S2). In all cases, *p*-values were equal or below 0.05.

The main conclusion is that sMDiNs can successfully be used as a basis for class discrimination not impairing and sometimes improving the performance of clustering or PLS-DA model classification and improving the classification by RF for all benchmark datasets. This was observed for hard problems as in the *GD* datasets (multiclass, low number of samples per class), easy problems as in *YD*, and two-class problems such as *GD types* and *HD* datasets. Therefore, there is useful and discriminatory information carried over and highlighted in sMDiNs, which makes its use in this context viable when compared to the workflows that start with two-dimensional numerical intensity matrices.

### Example of Feature Importance Assignment From sMDiNs

**FIGURE 5:**
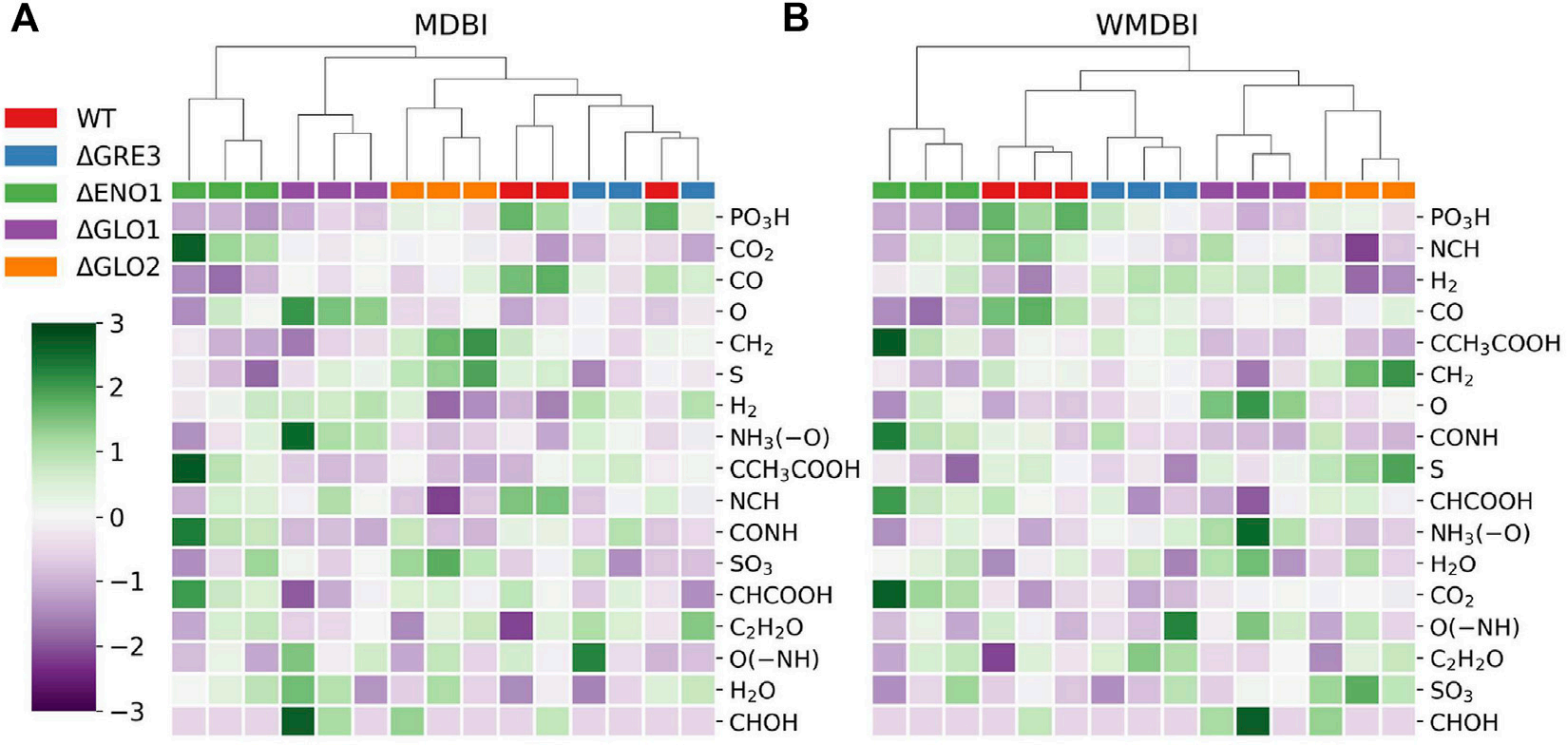
MDBI and WMDBI values for the sMDiNs of the *YD* dataset. **(A)** MDB impact; **(B)** weighted MDB impact. Values were mean-centered and standard scaled. MDBs are ordered by decreasing gini importance. Samples are triplicates of yeast strains of the wild-type reference strain (WT) and four single-gene deletion isogenic mutants of this strain: ΔGLO1, ΔGLO2, ΔGRE3, and ΔENO1. MDBs are listed in Table 2. Samples were clustered by HCA, with Euclidean distance and Ward linkage.

We next aimed to establish if the information extracted from sMDiNs might complement the type of information drawn using the traditional data analysis workflow. To this end, we assessed the use of MDBI and WMDBI to assign importance to the MDBs. These two metrics characterize each sMDiN based on the edges established by each of the MDBs and should then represent the prominence of the different types of reactions represented by them in the sample. Assessing which MDBs are more important for class discrimination might point to chemical transformations that are over- or under-represented in a subset of classes in the dataset. This concept is similar to the one used by Moritz and coworkers to identify and characterize differences of two gray poplar genotypes, although approached with a different methodology (Moritz et al., 2017). For example, if oxidizing compounds or enzymes are more present or expressed in a biological system when compared to others, the presence of more metabolites whose difference corresponds to an oxidation reaction (O or H_2_) is expected. For simplicity, so that the differences in the classes may be easily observable, the *YD* dataset was used as an example due to the smaller number of samples and classes. The importance of each of the MDBs estimated by their gini importance (Louppe et al., 2013) for building the RF models is shown in Table 4 and cluster maps of the data with decreasing rank of MDB importance are depicted in Figure 5.

**TABLE 4:** MDB gini importance for the RF model developed from the MDBI and WMDBI values for the *YD* dataset.

Rank	MDB impact		Weighted MDB impact	
	MDB	Gini importance	MDB	Gini importance
1	PO_3_H	0.080861	PO_3_H	0.074093
2	CO_2_	0.078512	NCH	0.071493
3	CO	0.075893	H_2_	0.069716
4	O	0.071955	CO	0.069039
5	CH_2_	0.069898	CCH_3_COOH	0.066709
6	S	0.068733	CH_2_	0.065887
7	H_2_	0.067228	O	0.065582
8	NH_3_(–O)	0.067118	CONH	0.065106
9	CCH_3_COOH	0.065289	S	0.063815
10	NCH	0.057191	CHCOOH	0.062894
11	CONH	0.054210	NH_3_(–O)	0.061970
12	SO_3_	0.053020	H_2_O	0.057914
13	CHCOOH	0.049574	CO_2_	0.054781
14	C2H2O	0.047399	O(–NH)	0.052759
15	O(–NH)	0.038924	C_2_H_2_O	0.048037
16	H_2_O	0.037026	SO_3_	0.040224
17	CHOH	0.017169	CHOH	0.009983

The MDB with the greatest impact for class classification in both metrics is “PO_3_H,” which represents metabolite phosphorylation and dephosphorylation (Table 4). Thus, this MDB seems fundamental to class discrimination. It has higher values in the WT strain, intermediate in ΔGRE3 and ΔGLO2, and lower in ΔENO1 and ΔGLO1 for both methods (Figure 5). Other MDBs follow the same pattern, such as CH_2_ (methylation) and CO (formylation) also ranking high. On the other hand, with the least important MDBs for both methods (CHOH or O(–NH) for example), there is no stratification of values per class and a lack of consistency among samples of the same class. MDB impacts can be interpreted as reactions being over- or under-represented in some strains, that is, for example, phosphorylation-related reactions being under-represented in the yeast mutant strains from the WT, especially in ΔENO1 and ΔGLO1. In contrast, the prevalence of reactions represented by the least important MDBs was not altered significantly by the single-gene deletions. This helps the characterization of the strains and may be used to guide future studies, for example, in this case, to justify the under-representation of phosphorylation reaction in the single-gene deletion mutants. The use of this approach in metabolomics data analysis can become useful in two-class problems where differential representation can be more easily observable.

The “carboxymethylation” and “carboxyethylation” MDBs were added specifically for the *YD* dataset because they are related to protein and phospholipid glycation, specific for phosphatidylethanolamines (Requena et al., 1997; Sousa Silva et al., 2013). Glycation is a post-translational modification resulting from the reaction of methylglyoxal with arginine and lysine residues (Sousa Silva et al., 2013). Despite glycation being mainly associated with proteins, carboxymethylated and carboxyethylated phospholipids, considered advanced glycation end-products, have been detected *in vivo*, with two main formation mechanisms proposed: direct transformation after reaction with glyoxal and methylglyoxal (carboxymethyl and ethyl, respectively) or indirectly after a set of different reactions (Requena et al., 1997; Shoji et al., 2010). It is expected that these MDBs should be more prevalent in the yeast strains with single-gene deletions, namely, ΔGLO1 and ΔGRE3, and in a lesser extent in ΔGLO2, as the deleted enzymes are directly involved in methylglyoxal catabolism, an anti-glycation mechanism defense (Sousa Silva et al., 2013). Although small differences can be observed between strains, neither of these MDBs ranked very high in importance. These results confirmed that when grown under normal culture conditions (without exposure to glycation), these mutants do not present a glycation phenotype (Gomes et al., 2006). This analysis shows the possible and untraditional types of information that can be highlighted using sMDiNs as a basis for data analysis. As a general note, node-centric and local characteristics analysis will probably shed information on individual or a set of nodes (*m/z* peaks), whereas global analysis will focus on the general and overall characteristics of the system.

## Discussion

The use of MDiNs as a basis for class discrimination and identification of important features relies on the commonly accepted concept that the detected metabolites identified with high-resolution methods are characteristic of a biological system. Here, the set of spectral features is used to build a mass-difference network. This representation, where each sample has a different MDiN, is a basis for downstream data analysis. This approach is suitable for untargeted metabolomics, although it requires high-resolution and high-accuracy mass-spectrometry—based data. As stated, sMDiNs are built *ab initio* by considering the detected metabolites as nodes with edges established if the difference in mass between nodes corresponds to one of the MDBs representing types of chemical transformations, generating networks akin to metabolic networks without *a priori* information regarding the system. The relevant characteristics of data here are how each detected feature might relate to other detected features. Therefore, unconnected nodes are uninformative.

Several high-resolution metabolomics datasets were used as benchmarks to test the hypothesis that data representing profiles of graph properties can be used for clustering and classification. As these datasets come from different sources and different instrumental platforms and workflows and have different levels of class overlap, number of samples, and classes, they are real-life scenarios of data quality levels in current high-resolution metabolomics.

### Advantages of Sample Mass-Difference Networks

This study indicates that using sample MDiNs as a data basis for class characterization and discrimination, instead of the usual two-dimensional numerical data matrices, is viable in metabolomics data analysis and capable of offering and highlighting complementary information to traditional workflows. Node-centric network metrics generated profiles that resulted in similar or better class discrimination in downstream statistical analysis methods in comparison to IDT, which represents traditional methods in this study. The network analyses of the sMDiNs are an alternative to the data pretreatments of numerical matrices. Perhaps the greatest advantage of sMDiNs is the versatility that it provides. Although all traditional pretreatments are alternatives with a common objective of highlighting relevant biological information contained within the data intensity values while reducing the effect of undesired variation (van den Berg et al., 2006), network analysis of sMDiNs can be oriented to highlight wildly different aspects of the data such as node centrality, network topology, edge properties, and metabolite taxonomy (in case the MDiNs are also used for formula assignment). These provide complementary information and can, therefore, be used in parallel and even articulated together. As an example, in this study, the degree analysis of the sMDiNs showed that they contain discriminatory information for class classification. Then, the importance of the nodes based on an RF model of these data was used to give weights to their corresponding edges to improve the MDBI analysis. With only 15 features, we could use this weighted MDBI to observe what type of chemical reactions were being over- or under-represented between the dataset’s classes by accessing their importance for building classifier models. Hence, it is possible to obtain multiple types of information from the metabolites that establish different connections between classes (degree) and what type of chemical reaction is differently represented in the classes (WMDBI). So far, we only scratched the surface testing only six network analysis methods because this preliminary study was focused on determining the viability of using sMDiN as a basis for class discrimination, and thus, a systematic study of different network analysis methods used was beyond the scope of the article. MDiNs might also be useful to compare samples from different datasets and experiments even in the absence of proper retention time or *m/z* alignment or formula assignment by falling back on comparing the global sMDiN characteristics such as MDBI that, although less efficient, may still provide meaningful insight for sample and class characterization. As discussed later, this versatility comes at the cost of some additional methodological complexity.

MDiNs consider all possible reaction interactions between spectral features based on the set of MDBs used and account for both enzymatic and nonenzymatic chemical reactions in the representation of the metabolome and its interactions. Apart from the *a priori* choice of MDBs, they are not restricted by prior knowledge of the different metabolic pathways that make up the metabolic networks, knowledge of which may still be very incomplete in less studied biological systems (Moritz et al., 2017). Furthermore, the derivation of an MDiN does not require metabolite identification, needed to map a dataset to metabolic networks. Thus, it can be applied to the study of any biological system.

The application of sMDiNs in this work discarded the intensity signals in the data to build the networks. Although we do not reject the idea of using the intensities as node information or even to help in building the networks, discarding these signals has some advantages (Traquete et al., 2021). This allows us to primarily use the occurrence of spectral features, which we have shown to be able to discriminate between biological systems (Traquete et al., 2021). These data should be less subjected to variability than intensity. For example, Lin and coworkers found that compound annotation was less variable in experiments between laboratories than relative quantification of those metabolites (Lin et al., 2020). Thus, this is useful as it diminishes inherent variability. Despite relying on the occurrence of spectral features and thus requiring the presence of a sufficient number of missing values, the methods based on sMDiNs even worked for the *HD* dataset, which had a lower number of missing values.

Network analysis of each sample’s sMDiN proceeds independently of the other samples (except for the WMDBI). It is, therefore, robust to data leakage in training–testing procedures: the possible introduction of feature distribution information, a pitfall of the traditional preprocessing procedures, does not happen with sMDiNs. Thus, for model validation, the care to apply the preprocessing pipeline to the train and test sets independently is not necessary.

Another advantage of sMDiNs is that they are already used for formula assignment (Tziotis et al., 2011; Moritz et al., 2017; Fudyma et al., 2019). Thus, feature annotation and formula assignment can be efficiently coupled with sMDiN property analysis, allowing metabolite identification to be used for data biological interpretation, but it may also motivate specific network analysis. For example, from the assigned formulas, compound taxonomy based on elemental ratios can be annotated (Rivas-Ubach et al., 2018), which, in turn, can be used to identify metabolite taxa over- or under-represented in the different classes, similar to what was demonstrated for MDBI analysis, but focusing on nodes instead of edges.

### General Applicability and Limitations of Sample Mass-Difference Networks

In this study, sMDiN application was benchmarked with examples of datasets obtained in different high-resolution MS instruments and with different number of samples, classes, and features, leading to good performances in the application of the clustering and classifier methods.

The derivation of sMDiNs from spectral data is difficult if these data are not obtained with high-resolution metabolomics instruments and methodologies. High resolution is usually associated with high mass accuracy, an instrumental capability that is paramount to establish accurate mass differences to reliably find the connections that correspond to a given MDB (Breitling et al., 2006; Ruf et al., 2018). In this work, a tolerance of 1 ppm for relative mass-difference error was employed. Moreover, a high number of features are necessary to allow for enough edges in the sMDiNs, meaning that the network density must be sufficiently high to characterize each sample. The usage of sMDiNs based on the difference in the set of features detected in each sample is grounded on the idea that every metabolome will have its own set of metabolites as a key information source for the discrimination. This specificity is reflected in the specificity of network properties. Thus, the differential occurrence of spectral features is essential with the disparity between missing and nonmissing values becoming important for MDiN building. sMDiN based analysis might become unsuitable if data have a very low or very high number of missing values. High-resolution datasets have considerable missing-value abundance as opposed to lower resolution data with lower amounts of missing values due to broad bins/peaks. Missing values are usually excluded by variable selection based on threshold of missing-value abundance, and their importance in discrimination is overlooked and overshadowed by intensity data. Here, the information content of missing values is fully used to contrast the different sMDiNs. In this study, feature selection was mild and resulted from the application of a feature filtering procedure based on a reproducibility criterion to exclude features that only appeared once in the set of all samples, most of which would be uninformative being the result of noise or variability. For dataset *GDc2*, features that did not appear in at least two samples of the same class were excluded (a stricter criterion).

The construction of each sMDiN from direct infusion data is not able to incorporate differences in isobaric compounds or functionally different isomers, and each edge in the network could arise from any of the multiple possible specific reactions. Chromatographic separation data can, in principle, inform on the chemical structure behind a given mass value and can help in curating the network, allowing for the separation of nodes with the same mass. This cannot, however, be performed in general for most nodes of the sMDiNs, and the structural changes in the networks are not expected to be significant after the incorporation of retention time information. Even without using a finer network structure in our direct benchmark datasets, we were able to demonstrate that the graph metrics carry enough information to make clustering or classification procedures successful.

Mass-difference network construction requires the selection of the error allowed for MDBs, and the choice of an MDBs list to consider is crucial. MDBs should encompass transformations that affect at least the most common elements in metabolites (C, H, O, N, S, and P) and should have some basis on observed chemical reactions. They can be chosen *a priori* from a list of predefined masses or by the most common mass differences present in the dataset capable of being assigned a specific chemical transformation (Kunenkov et al., 2009; Moritz et al., 2017). MDBs may establish edges not corresponding to their intended chemical transformation but to a set of smaller reactions that have cumulatively the same elemental changes and mass differences. For example, transfer of a formyl group (change of CO) plus hydrogenation (H_2_ change) leads to the same change of hydroxymethylation (change of CHOH). Furthermore, specific sets of chemical transformations may be added based on the biological systems analyzed and the purpose of the experiment. Thus, the number of MDBs and the tolerance for the mass-difference error allowed in MDiN construction should also be set based on the particular datasets and instrumental capabilities to prevent the sMDiNs to become too dense (their topology becomes too similar among the different samples) or too sparse (where not enough connections are established to differentiate graph properties). However, the choice of MDBs for a specific purpose must take into account the possibility of in-source dissociation of some compounds, leading, for example, to water loss, which corresponds to a dehydration MDB. In this work, we chose to restrict to only 15 MDBs (Table 2) as a proof of concept. The MDBs chosen represent small and ubiquitous reactions in biological systems that we believe should be considered in most MDiNs of biological samples. Addition of other MDBs might be necessary in other untargeted analytical contexts, such as environmental studies, where a more in-depth analysis of the chemical complexity is required, considering previous knowledge of the systems under study and the experimental objective.

With the parameter-tuning of mass error tolerance and choice of MDBs, primary MDiNs, like the ones in this study, can be derived. Extra steps may be necessary to increase the quality of the sMDiNs. For example, false positive edges can be established between two masses whose MDB does not correspond to their actual differences in elemental compositions. This is more likely to happen with higher masses, where the combination of possible formulas within a 1 ppm error margin increases exponentially (Kind and Fiehn, 2006). This can generate spurious connections between metabolites. To tackle this issue, the fact that MDiNs have been used to assign formulas to detected peaks (Breitling et al., 2006; Moritz et al., 2017), starting from a set of reliable annotated metabolites (higher confidence formulas) and propagating them through the network components can be of use. With this formula assignment, besides adding information to the network nodes, we can see if discrepancies arise in formula propagation from different starting nodes in a component, which can help in flagging possible spurious connections. Furthermore, imposing elemental ratio constraints, for example, the number of oxygen atoms to number of carbon atoms ratio (Kind and Fiehn, 2007), may help in assigning formulas within the allowed regions of the chemical space, while removing edges that would lead to formulas outside those regions. There can also be instances where very similar masses can be linked by the same MDB in the same “direction” (addition or subtraction) to the same node (as shown in the inset of Figure 2, where nodes 255.2927 and 255.2925 were both linked to node 299.2924 by a CO_2_ edge) and would represent metabolites with the same elemental formula. This could be the result of errors in the peak selection or alignment stages or because the mass error tolerance for building the MDiN was too high, for example. A possible solution would be to eliminate the edge with a higher associated error or to reduce error tolerance. Here, we demonstrate that it is possible for sMDiNs to be useful for class discrimination with high-quality and resolution metabolomics datasets with a simplistic application using a restricted list of MDBs, even without taking measures to increase sMDiN quality, showing the base potential of the methodology.

The next step is to carefully choose and apply different network analysis. Careful deliberation by the researcher regarding the network analysis methods that will be employed should be made because the information that they provide can be very specific, whether focused on node characteristics, local clusters, and edge properties such as MDBI or node properties like compound taxonomy. Thus, this methodology keeps class discrimination information while remaining versatile in the types of information that it can provide, which can be specific to the intention and information desired from the experimental work. Nevertheless, for larger datasets such as *HD*, some network analyses of a high number of samples might be computationally prohibitive.

MDiNs lead to a very simple type of feature selection: rejection of unconnected, uninformative nodes in the MDiN. These tend to be approximately half to two-thirds of the dataset, resulting in a hefty filter process. This rejection retains features that (based on the MDBs selected) connect to at least another detected metabolite in the dataset. The possible side effect is the removal of some rarer and unorthodox metabolites that may exist and be informative. Further feature selection should not be made because every remaining node has an impact on the full dataset because it affects the network property values of other nodes. Thus, there are no counterparts to the types of feature selection normally used (statistical significance, classifier feature importance, or number of missing values). Other feature selection procedures prior to building MDiNs should be carefully applied as to not exclude too many features as well as to not exclude informative features with plenty of missing values.

## Conclusion

The adoption of MDiNs at a sample level (sMDiNs) in the metabolomics data analysis framework was envisioned to be a viable alternative to the analysis of two-dimensional numerical matrices for biological system characterization and discrimination. MDiNs are built using the list of detected features (*m/z* peaks) as nodes, establishing edges between nodes if their difference in mass corresponds to a chosen list of allowed chemical transformations that can occur in biological systems. Thus, they depict the chemical diversity of a sample building a network akin to a metabolic network, without the need for metabolite identification and mapping to the (usually incomplete) known metabolic pathways. Sample MDiNs were built based on the features detected for each sample. Thus, differences between samples come from the difference in the set of features detected that dictate the nodes, edges, and other network properties to be analyzed, contrasting with the use of signal intensities by the two-dimensional numerical matrices. Graph property network analysis of the sMDiNs is a novel idea that we present as an alternative to data pretreatments but with the added benefit of being able to focus on different characteristics of the networks.

The four statistical methods employed performed consistently as well or slightly better with data from the degree analysis of sMDiNs than with data treated with common intensity-based pretreatments, showing that discriminatory information is retained in the sMDiNs. The use of a different network analysis for important feature assignment was also shown by highlighting weighted MDBI analysis on yeast data sMDiNs that revealed an under-representation of phosphorylation-like reactions in the single-gene deletion yeast mutants compared to the WT as an example of the possible versatility of the methodology.

The procedure to build sMDiNs requires a high number of features and high-resolution data as well as a balanced missing-value occurrence. Thus, it is not generalizable to all scenarios for metabolomics data analysis. It can be efficiently coupled with formula assignment and feature selection methodologies based on the MDiN concept, which offers extra information for class discrimination and biological interpretation.

On the basis of the demonstrated viability of the procedure as well as the additional information that can be gained from it, we propose the use of sMDiNs as a basis for the analysis of high-resolution untargeted data in metabolomics.

## Data Availability

Publicly available datasets were analyzed in this study. These data can be found here: https://figshare.com/articles/dataset/Grapevine_untargeted_metabolomics_to_uncover_potential_biomarkers_of_fungal_oomycetes-associated_diseases/12357314, https://figshare.com/articles/dataset/FT-ICR-MS_based_untargeted_metabolomics_for_the_discrimination_of_yeast_mutants/15173559, https://www.metabolomicsworkbench.org/data/DRCCMetadata.php?Mode=Project&ProjectID=PR000724.

## Abbreviations

AUC, area under the curve; FT-ICR-MS, Fourier transform ion-cyclotron resonance mass spectrometry; GCD-11/GCD11, graphlet correlation distance that includes the 11 nonredundant orbits of up to four-node graphlets; *GD*, grapevine dataset; *GDc2*, grapevine dataset retaining features that occur globally at least twice in at least one class; *GDg2*, grapevine dataset retaining features that occur globally at least twice in the dataset; HCA, agglomerative hierarchical clustering analysis; *HD*, human dataset; HILIC, hydrophilic interaction chromatography; IDT, intensity-based data pretreatment; MDBI, mass-difference-based building blocks impact; MDBs, mass-difference-based building blocks; MDiNs, mass-difference networks; MS, mass spectrometry; PLS, projection in latent structures (least squares); PLS-DA, projection in latent structures–discriminant analysis; RF, random forest; sMDiN, sample mass-difference network; WMDBI, weighted mass-difference-based building blocks impact; *YD*, yeast dataset.

## References

[B1] Amara A. Frainay C. Jourdan F. Naake T. Neumann S. Novoa-del-Toro E. M. (2022). Networks and Graphs Discovery in Metabolomics Data Analysis and Interpretation. Front. Mol. Biosci. 9. 10.3389/fmolb.2022.841373 PMC8957799 35350714

[B2] Andreopoulos B. An A. Wang X. Schroeder M. (2009). A Roadmap of Clustering Algorithms: Finding a Match for a Biomedical Application. Briefings Bioinforma. 10, 297–314. 10.1093/bib/bbn058 19240124

[B3] Barabási A.-L. Oltvai Z. N. (2004). Network Biology: Understanding the Cell's Functional Organization. Nat. Rev. Genet. 5, 101–113. 10.1038/nrg1272 14735121

[B4] Bartel J. Krumsiek J. Theis F. J. (2013). Statistical Methods for the Analysis of High-Throughput Metabolomics Data. Comput. Struct. Biotechnol. J. 4, e201301009. 10.5936/csbj.201301009 24688690 PMC3962125

[B5] Breitling R. Ritchie S. Goodenowe D. Stewart M. L. Barrett M. P. (2006). Ab Initio prediction of Metabolic Networks Using Fourier Transform Mass Spectrometry Data. Metabolomics 2, 155–164. 10.1007/s11306-006-0029-z 24489532 PMC3906711

[B6] Burgess K. E. V. Borutzki Y. Rankin N. Daly R. Jourdan F. (2017). MetaNetter 2: A Cytoscape Plugin for Ab Initio Network Analysis and Metabolite Feature Classification. J. Chromatogr. B 1071, 68–74. 10.1016/j.jchromb.2017.08.015 PMC5726607 29030098

[B7] Clancy M. V. Zytynska S. E. Moritz F. Witting M. Schmitt-Kopplin P. Weisser W. W. (2018). Metabotype Variation in a Field Population of Tansy Plants Influences Aphid Host Selection. Plant. Cell Environ. 41, 2791–2805. 10.1111/pce.13407 30035804

[B8] Clendinen C. S. Gaul D. A. Monge M. E. Arnold R. S. Edison A. S. Petros J. A. (2019). Preoperative Metabolic Signatures of Prostate Cancer Recurrence Following Radical Prostatectomy. J. Proteome Res. 18, 1316–1327. 10.1021/acs.jproteome.8b00926 30758971

[B9] Cuevas-Delgado P. Dudzik D. Miguel V. Lamas S. Barbas C. (2020). Data-dependent Normalization Strategies for Untargeted Metabolomics-A Case Study. Anal. Bioanal. Chem. 412, 6391–6405. 10.1007/s00216-020-02594-9 32285184

[B10] Dieterle F. Ross A. Schlotterbeck G. Senn H. (2006). Probabilistic Quotient Normalization as Robust Method to Account for Dilution of Complex Biological Mixtures. Application in 1H NMR Metabonomics. Anal. Chem. 78, 4281–4290. 10.1021/ac051632c 16808434

[B11] Ferreira A. E. N. Traquete F. (2021). Metabolinks: a Python Package for High-Resolution-MS Metabolomics Data Analysis. 10.5281/ZENODO.5336951

[B12] Forcisi S. Moritz F. Lucio M. Lehmann R. Stefan N. Schmitt-Kopplin P. (2015). Solutions for Low and High Accuracy Mass Spectrometric Data Matching: A Data-Driven Annotation Strategy in Nontargeted Metabolomics. Anal. Chem. 87, 8917–8924. 10.1021/acs.analchem.5b02049 26197019

[B13] Fudyma J. D. Lyon J. AminiTabrizi R. Gieschen H. Chu R. K. Hoyt D. W. (2019). Untargeted Metabolomic Profiling of Sphagnum Fallax Reveals Novel Antimicrobial Metabolites. Plant Direct 3, 1–17. 10.1002/pld3.179 PMC6848953 31742243

[B14] Gomes R. A. Miranda H. V. Sousa Silva M. Graça G. Coelho A. V. Ferreira A. E. (2006). Yeast Protein Glycationin Vivoby Methylglyoxal. Molecular Modification of Glycolytic Enzymes and Heat Shock Proteins. FEBS J. 273, 5273–5287. 10.1111/j.1742-4658.2006.05520.x 17064314

[B15] Gromski P. S. Muhamadali H. Ellis D. I. Xu Y. Correa E. Turner M. L. (2015). A Tutorial Review: Metabolomics and Partial Least Squares-Discriminant Analysis - a Marriage of Convenience or a Shotgun Wedding. Anal. Chim. Acta 879, 10–23. 10.1016/j.aca.2015.02.012 26002472

[B16] Hagberg A. A. Schult D. A. Swart P. J. (2008). “Exploring Network Structure, Dynamics, and Function Using NetworkX,” in Proceedings of the 7th Python in Science Conference, Austin, Texas, June 28 - July 30, 2008. Editors Varoquaux G. Vaught T. Millman J., 11–15.

[B17] Harris C. R. Millman K. J. van der Walt S. J. Gommers R. Virtanen P. Cournapeau D. (2020). Array Programming with NumPy. Nature 585, 357–362. 10.1038/s41586-020-2649-2 32939066 PMC7759461

[B18] Hunter J. D. (2007). Matplotlib: A 2D Graphics Environment. Comput. Sci. Eng. 9, 90–95. 10.1109/MCSE.2007.55

[B19] Jeske L. Placzek S. Schomburg I. Chang A. Schomburg D. (2019). BRENDA in 2019: a European ELIXIR Core Data Resource. Nucleic Acids Res. 47, D542–D549. 10.1093/nar/gky1048 30395242 PMC6323942

[B20] Johnson C. H. Gonzalez F. J. (2012). Challenges and Opportunities of Metabolomics. J. Cell. Physiol. 227, 2975–2981. 10.1002/jcp.24002 22034100 PMC6309313

[B21] Kaling M. Schmidt A. Moritz F. Rosenkranz M. Witting M. Kasper K. (2018). Mycorrhiza-Triggered Transcriptomic and Metabolomic Networks Impinge on Herbivore Fitness. Plant Physiol. 176, 2639–2656. 10.1104/pp.17.01810 29439210 PMC5884605

[B22] Karaman I. (2017). Preprocessing and Pretreatment of Metabolomics Data for Statistical Analysis. Adv. Exp. Med. Biol. 965, 145–161. 10.1007/978-3-319-47656-8_6 28132179

[B23] Katajamaa M. Orešič M. (2007). Data Processing for Mass Spectrometry-Based Metabolomics. J. Chromatogr. A 1158, 318–328. 10.1016/j.chroma.2007.04.021 17466315

[B24] Kind T. Fiehn O. (2006). Metabolomic Database Annotations via Query of Elemental Compositions: Mass Accuracy Is Insufficient Even at Less Than 1 Ppm. BMC Bioinforma. 7, 234. 10.1186/1471-2105-7-234 PMC1464138 16646969

[B25] Kind T. Fiehn O. (2007). Seven Golden Rules for Heuristic Filtering of Molecular Formulas Obtained by Accurate Mass Spectrometry. BMC Bioinforma. 8, 1–20. 10.1186/1471-2105-8-105 PMC1851972 17389044

[B26] Kokla M. Virtanen J. Kolehmainen M. Paananen J. Hanhineva K. (2019). Random Forest-Based Imputation Outperforms Other Methods for Imputing LC-MS Metabolomics Data: a Comparative Study. BMC Bioinforma. 20, 492. 10.1186/s12859-019-3110-0 PMC6788053 31601178

[B27] Kunenkov E. V. Kononikhin A. S. Perminova I. V. Hertkorn N. Gaspar A. Schmitt-Kopplin P. (2009). Total Mass Difference Statistics Algorithm: A New Approach to Identification of High-Mass Building Blocks in Electrospray Ionization Fourier Transform Ion Cyclotron Mass Spectrometry Data of Natural Organic Matter. Anal. Chem. 81, 10106–10115. 10.1021/ac901476u 19904912

[B28] Laber S. Forcisi S. Bentley L. Petzold J. Moritz F. Smirnov K. S. (2021). Linking the FTO Obesity Rs1421085 Variant Circuitry to Cellular, Metabolic, and Organismal Phenotypes In Vivo. Sci. Adv. 7, 1–23. 10.1126/sciadv.abg0108 PMC8294759 34290091

[B29] Lee L. C. Liong C.-Y. Jemain A. A. (2018). Partial Least Squares-Discriminant Analysis (PLS-DA) for Classification of High-Dimensional (HD) Data: A Review of Contemporary Practice Strategies and Knowledge Gaps. Analyst 143, 3526–3539. 10.1039/c8an00599k 29947623

[B30] Lee S. Oh D.-G. Singh D. Lee J. S. Lee S. Lee C. H. (2020). Exploring the Metabolomic Diversity of Plant Species across Spatial (Leaf and Stem) Components and Phylogenic Groups. BMC Plant Biol. 20, 39. 10.1186/s12870-019-2231-y 31992195 PMC6986006

[B31] Lin Y. Caldwell G. W. Li Y. Lang W. Masucci J. (2020). Inter-laboratory Reproducibility of an Untargeted Metabolomics GC-MS Assay for Analysis of Human Plasma. Sci. Rep. 10, 1–11. 10.1038/s41598-020-67939-x 32616798 PMC7331679

[B32] Liu Y. Forcisi S. Harir M. Deleris-Bou M. Krieger-Weber S. Lucio M. (2016). New Molecular Evidence of Wine Yeast-Bacteria Interaction Unraveled by Non-targeted Exometabolomic Profiling. Metabolomics 12, 69. 10.1007/s11306-016-1001-1

[B33] Louppe G. Wehenkel L. Sutera A. Geurts P. (2013). Understanding Variable Importances in Forests of Randomized Trees. Adv. Neural Inf. Process. Syst. 26, 431–439.

[B34] Luz J. (2021). Metabolomic Effects of Single Gene Deletions in Saccharomyces cerevisiae. Master Thesis in Biochemistry. Lisboa, Portugal: Faculdade de Ciências da Universidade de Lisboa.

[B35] Luz J. Pendão A. S. Sousa Silva M. Cordeiro C. (2021). FT-ICR-MS Based Untargeted Metabolomics for the Discrimination of Yeast Mutants. figshare. Dataset. 10.6084/m9.figshare.15173559.v1

[B36] Maia M. Ferreira A. E. N. Nascimento R. Monteiro F. Traquete F. Marques A. P. (2020a). Integrating Metabolomics and Targeted Gene Expression to Uncover Potential Biomarkers of Fungal/oomycetes-Associated Disease Susceptibility in Grapevine. Sci. Rep. 10–15. 10.1038/s41598-020-72781-2 PMC7515887 32973337

[B37] Maia M. Figueiredo A. Sousa Silva M. Ferreira A. (2020b). Grapevine Untargeted Metabolomics to Uncover Potential Biomarkers of Fungal/oomycetes-Associated Diseases. figshare. Dataset. 10.6084/m9.figshare.12357314.v2

[B38] McKinney W. (2010). “Data Structures for Statistical Computing in Python,” in Proceedings of the 9th Python in Science Conference, Austin, Texas, June 28 - 30, 2010. Editors van der Walt S. Millman J., 56–61. 10.25080/Majora-92bf1922-00a

[B39] Milenković T. Pržulj N. (2008). Uncovering Biological Network Function via Graphlet Degree Signatures. Cancer Inf. 6, CIN.S680–273. 10.4137/cin.s680 PMC2623288 19259413

[B40] Moritz F. Hemmler D. Kanawati B. Schnitzler J.-P. Schmitt-Kopplin P. (2019), Mass Differences in Metabolome Analyses of Untargeted Direct Infusion Ultra-high Resolution MS Data. Fundamentals and Applications of Fourier Transform Mass Spectrometry 2019, 357–405. 10.1016/B978-0-12-814013-0.00012-0

[B41] Moritz F. Janicka M. Zygler A. Forcisi S. Kot-Wasik A. Kot J. (2015). The Compositional Space of Exhaled Breath Condensate and its Link to the Human Breath Volatilome. J. Breath. Res. 9, 027105. 10.1088/1752-7155/9/2/027105 25944811

[B42] Moritz F. Kaling M. Schnitzler J.-P. Schmitt-Kopplin P. (2017). Characterization of Poplar Metabotypes via Mass Difference Enrichment Analysis. Plant, Cell & Environ. 40, 1057–1073. 10.1111/pce.12878 27943315

[B43] Morreel K. Saeys Y. Dima O. Lu F. Van de Peer Y. Vanholme R. (2014). Systematic Structural Characterization of Metabolites in Arabidopsis via Candidate Substrate-Product Pair Networks. Plant Cell 26, 929–945. 10.1105/tpc.113.122242 24685999 PMC4001402

[B44] Pedregosa F. Varoquaux G. Gramfort A. Michel V. Thirion B. Grisel O. (2011). Scikit-learn: Machine Learning in Python. J. Mach. Learn. Res. 12, 2825–2830.

[B45] Ramirez-Gaona M. Marcu A. Pon A. Guo A. C. Sajed T. Wishart N. A. (2017). YMDB 2.0: a Significantly Expanded Version of the Yeast Metabolome Database. Nucleic Acids Res. 45, D440–D445. 10.1093/nar/gkw1058 27899612 PMC5210545

[B46] Requena J. R. Ahmed M. U. Fountain C. W. Degenhardt T. P. Reddy S. Perez C. (1997). Carboxymethylethanolamine, a Biomarker of Phospholipid Modification during the Maillard Reaction In Vivo. J. Biol. Chem. 272, 17473–17479. 10.1074/jbc.272.28.17473 9211892

[B47] Rivas-Ubach A. Liu Y. Bianchi T. S. Tolić N. Jansson C. Paša-Tolić L. (2018). Moving beyond the Van Krevelen Diagram: A New Stoichiometric Approach for Compound Classification in Organisms. Anal. Chem. 90, 6152–6160. 10.1021/acs.analchem.8b00529 29671593

[B48] Roberts L. D. Souza A. L. Gerszten R. E. Clish C. B. (2012). Targeted Metabolomics. Curr. Protoc. Mol. Biol. 98, 30. 10.1002/0471142727.mb3002s98 PMC3334318 22470063

[B49] Roessner U. Hansen M. A. E. (2007). The Chemical Challenge of the Metabolome. Metabolome Anal., 15–38. 10.1002/9780470105511.ch2

[B50] Ruf A. d’Hendecourt L. Schmitt-Kopplin P. (2018). Data-Driven Astrochemistry: One Step Further within the Origin of Life Puzzle. Life 8, 18. 10.3390/life8020018 PMC6027145 29857564

[B51] Schmitt-Kopplin P. Hemmler D. Moritz F. Gougeon R. D. Lucio M. Meringer M. (2019). Systems Chemical Analytics: Introduction to the Challenges of Chemical Complexity Analysis. Faraday Discuss. 218, 9–28. 10.1039/c9fd00078j 31317165

[B52] Shannon P. Markiel A. Ozier O. Baliga N. S. Wang J. T. Ramage D. (2003). Cytoscape: a Software Environment for Integrated Models of Biomolecular Interaction Networks. Genome Res. 13, 2498–2504. 10.1101/gr.1239303 14597658 PMC403769

[B53] Shoji N. Nakagawa K. Asai A. Fujita I. Hashiura A. Nakajima Y. (2010). LC-MS/MS Analysis of Carboxymethylated and Carboxyethylated Phosphatidylethanolamines in Human Erythrocytes and Blood Plasma. J. Lipid Res. 51, 2445–2453. 10.1194/jlr.D004564 20386060 PMC2903796

[B54] Sousa Silva M. Gomes R. A. Ferreira A. E. Ponces Freire A. Cordeiro C. (2013). The Glyoxalase Pathway: the First Hundred years. And beyond. Biochem. J. 453, 1–15. 10.1042/BJ20121743 23763312

[B55] Sousa Silva M. Luz J. Pendão A. S. Cordeiro C. (2021). Magnetic Resonance Mass Spectrometry (MRMS) Discriminates Yeast Mutants through Metabolomics and Analysis. Bruker Appl. Note MRMS 75.

[B56] Stekhoven D. J. Bühlmann P. (2012). MissForest--non-parametric Missing Value Imputation for Mixed-type Data. Bioinformatics 28, 112–118. 10.1093/bioinformatics/btr597 22039212

[B57] Tantardini M. Ieva F. Tajoli L. Piccardi C. (2019). Comparing Methods for Comparing Networks. Sci. Rep. 9, 1–19. 10.1038/s41598-019-53708-y 31772246 PMC6879644

[B58] Traquete F. Luz J. Cordeiro C. Sousa Silva M. Ferreira A. E. N. (2021). Binary Simplification as an Effective Tool in Metabolomics Data Analysis. Metabolites 11, 788. 10.3390/metabo11110788 34822446 PMC8621519

[B59] Tziotis D. Hertkorn N. Schmitt-Kopplin P. (2011). Kendrick-analogous Network Visualisation of Ion Cyclotron Resonance Fourier Transform Mass Spectra: Improved Options for the Assignment of Elemental Compositions and the Classification of Organic Molecular Complexity. Eur. J. Mass Spectrom. (Chichester) 17, 415–421. 10.1255/ejms.1135 22006638

[B60] van den Berg R. A. Hoefsloot H. C. Westerhuis J. A. Smilde A. K. van der Werf M. J. (2006). Centering, Scaling, and Transformations: Improving the Biological Information Content of Metabolomics Data. BMC Genomics 7, 142. 10.1186/1471-2164-7-142 16762068 PMC1534033

[B61] Vinaixa M. Samino S. Saez I. Duran J. Guinovart J. J. Yanes O. (2012). A Guideline to Univariate Statistical Analysis for LC/MS-based Untargeted Metabolomics-Derived Data. Metabolites 2, 775–795. 10.3390/metabo2040775 24957762 PMC3901240

[B62] Virtanen P. Gommers R. Oliphant T. E. Haberland M. Reddy T. Cournapeau D. (2020). SciPy 1.0: Fundamental Algorithms for Scientific Computing in Python. Nat. Methods 17, 261–272. 10.1038/s41592-019-0686-2 32015543 PMC7056644

[B63] Walker A. Pfitzner B. Neschen S. Kahle M. Harir M. Lucio M. (2014). Distinct Signatures of Host-Microbial Meta-Metabolome and Gut Microbiome in Two C57BL/6 Strains under High-Fat Diet. ISME J. 8, 2380–2396. 10.1038/ismej.2014.79 24906017 PMC4260703

[B64] Waskom M. Botvinnik O. Gelbart M. Ostblom J. Hobson P. Lukauskas S. (2020). Mwaskom/Seaborn: v0.11.0 (Sepetmber 2020). 10.5281/zenodo.4019146

[B65] Weber R. J. M. Viant M. R. (2010). MI-pack: Increased Confidence of Metabolite Identification in Mass Spectra by Integrating Accurate Masses and Metabolic Pathways. Chemom. Intelligent Laboratory Syst. 104, 75–82. 10.1016/j.chemolab.2010.04.010

[B66] Wei R. Wang J. Su M. Jia E. Chen S. Chen T. (2018). Missing Value Imputation Approach for Mass Spectrometry-Based Metabolomics Data. Sci. Rep. 8, 1–10. 10.1038/s41598-017-19120-0 29330539 PMC5766532

[B67] Willkommen D. Lucio M. Moritz F. Forcisi S. Kanawati B. Smirnov K. S. (2018). Metabolomic Investigations in Cerebrospinal Fluid of Parkinson's Disease. PLoS One 13, e0208752. 10.1371/journal.pone.0208752 30532185 PMC6287824

[B68] Wishart D. S. Feunang Y. D. Marcu A. Guo A. C. Liang K. Vázquez-Fresno R. (2018). HMDB 4.0: The Human Metabolome Database for 2018. Nucleic Acids Res. 46, D608–D617. 10.1093/nar/gkx1089 29140435 PMC5753273

[B69] Worley B. Powers R. (2012). Multivariate Analysis in Metabolomics. Cmb 1, 92–107. 10.2174/2213235X11301010092 PMC4465187 26078916

[B70] Yaveroğlu Ö. N. Malod-Dognin N. Davis D. Levnajic Z. Janjic V. Karapandza R. (2014). Revealing the Hidden Language of Complex Networks. Sci. Rep. 4, 4547. 10.1038/srep04547 24686408 PMC3971399

