# Supplementary material for: Graph Properties of Mass-Difference Networks for Profiling and Discrimination in Untargeted Metabolomics
Source: Front Mol Biosci. 2022 Jul 22;9:917911. doi: 10.3389/fmolb.2022.917911 (PMC9353772; doi:10.3389/fmolb.2022.917911)
Supplement: Supplementary file 2 [file DataSheet1.pdf]

Supplementary Material

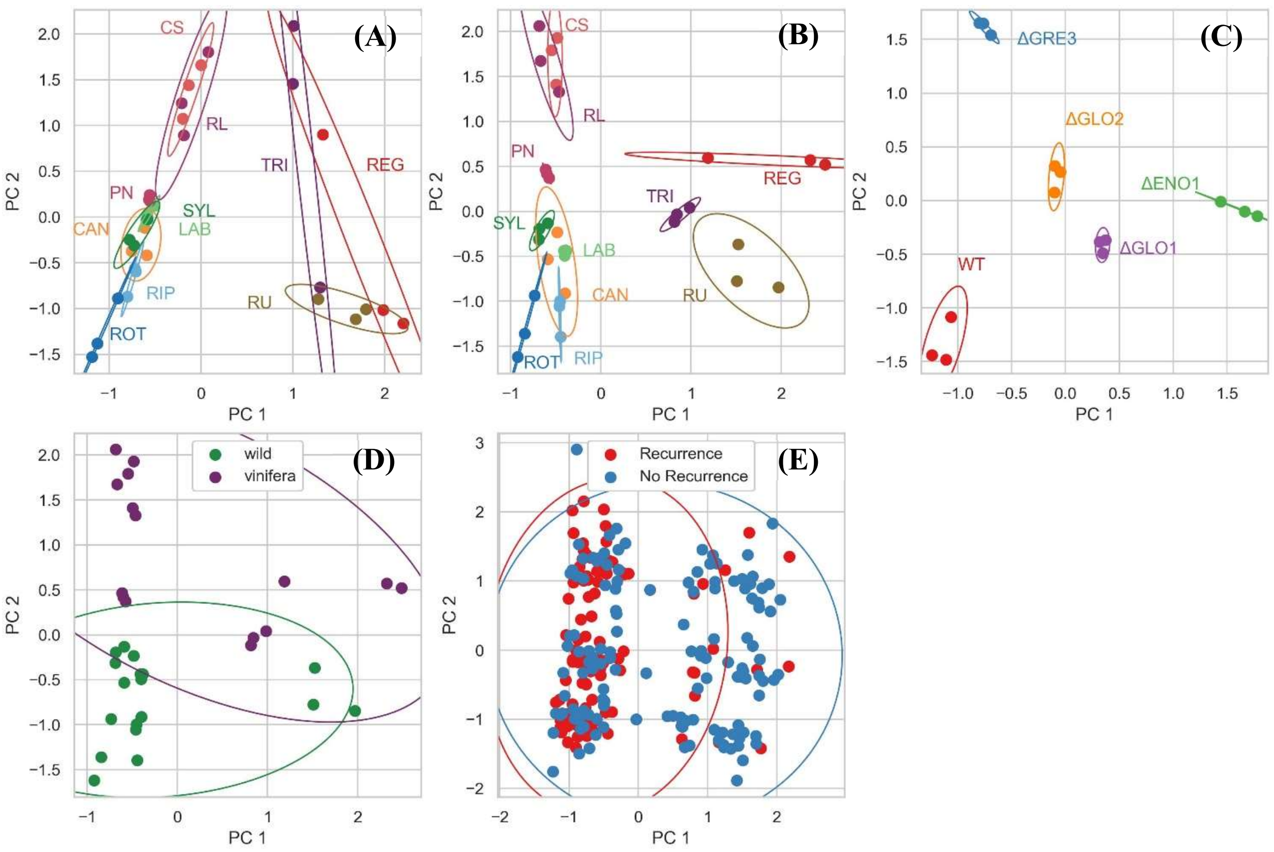

Supplementary Figure 1. Principal Component Analysis scores plots for the benchmark datasets. **(A)** *GDg2*; **(B)** *GDc2*; **(C)** *YD*; **(D)** *GD types*; **(E)** *HD*; All datasets were pre-treated with missing value imputation equal to 1/5th of the minimum of the non-missing values in each feature in the data matrix and standard-scaled. Labels identify replicates of each of the classes defined for the dataset. Ellipses are 95% confidence ellipses for each class.

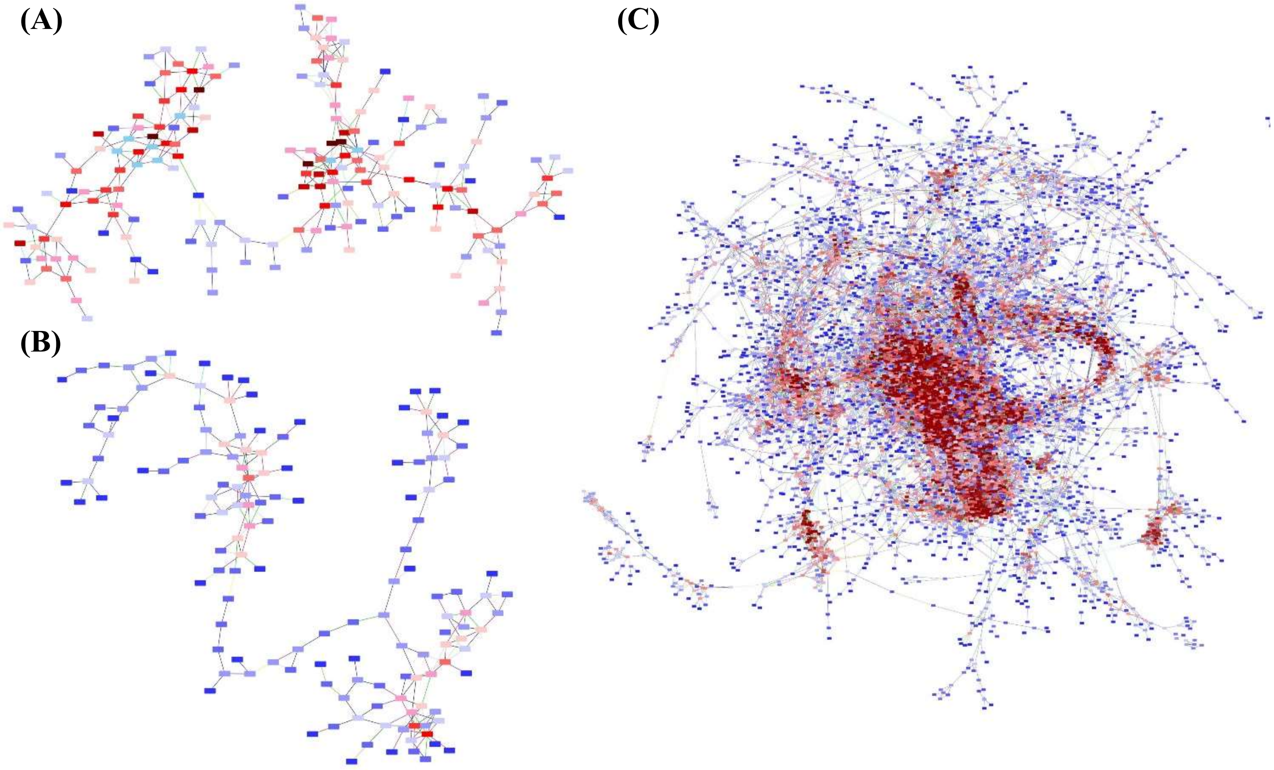

Supplementary Figure 2. Largest components of the Mass-Difference Networks constructed from the benchmark datasets. Datasets were **(A)** *GDg2*, **(B)** *GDc2* (and *GD types*) **(C)** *HD*. Node colour changes from blue (●) to dark red (●) with increasing degree. Edge colour represents the MDB (representing a set of chemical reactions) used to establish a connection: (—) – O(–NH), (—) – NH_3(–O), (—) – H_2, (—) – CH_2, (—) – O, (—) – H_2O, (—) – NCH, (—) – CO, (—) – CHOH, (—) – S, (—) – CH_2O, (—) – CONH, (—) – CO_2, (—) – SO_3, (—) – PO_3H. Network representations were made with the Cytoscape 3.8.1.

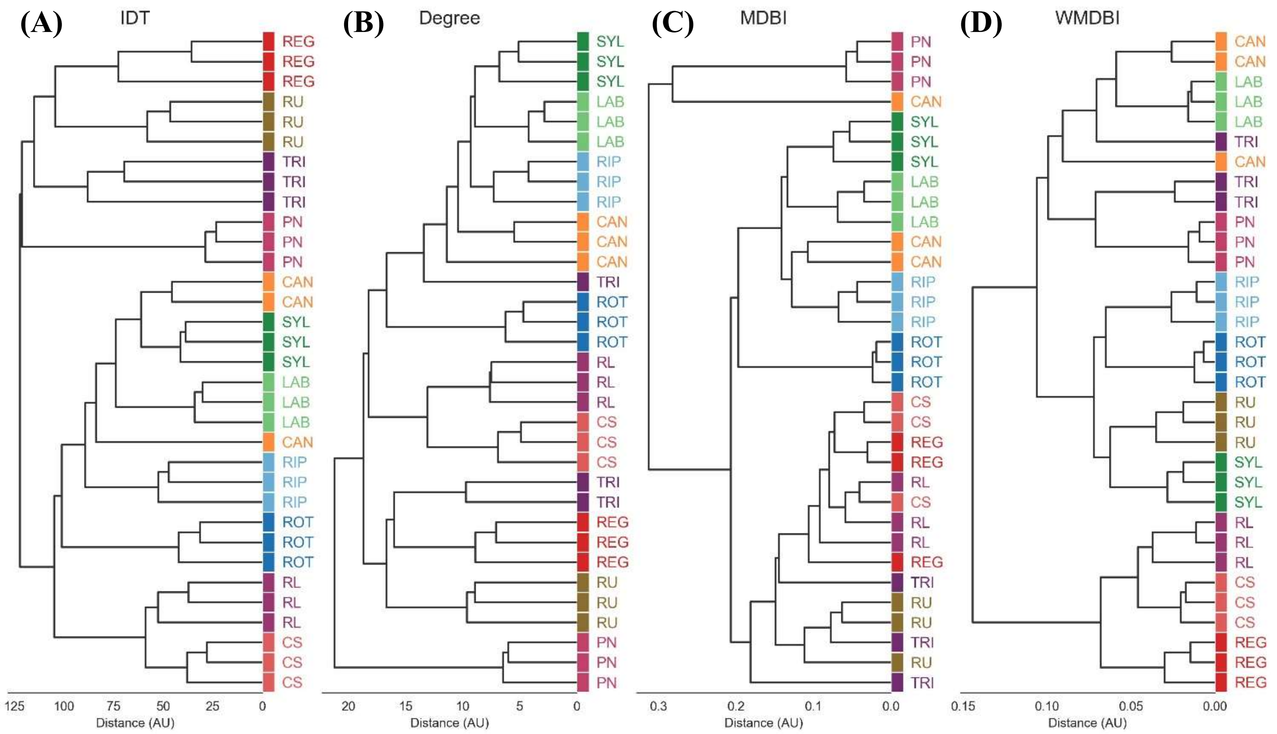

Supplementary Figure 3. Dendrograms resulting from the application of HCA to dataset *GDc2* after treatment with different methods. **(A)** IDT; **(B)** Degree analysis of sMDiNs; **(C)** MDB Impact analysis of sMDiNs; **(D)** Weighted MDB Impact analysis of sMDiNs. UPGMA linkage and Euclidean distance was used in all cases.

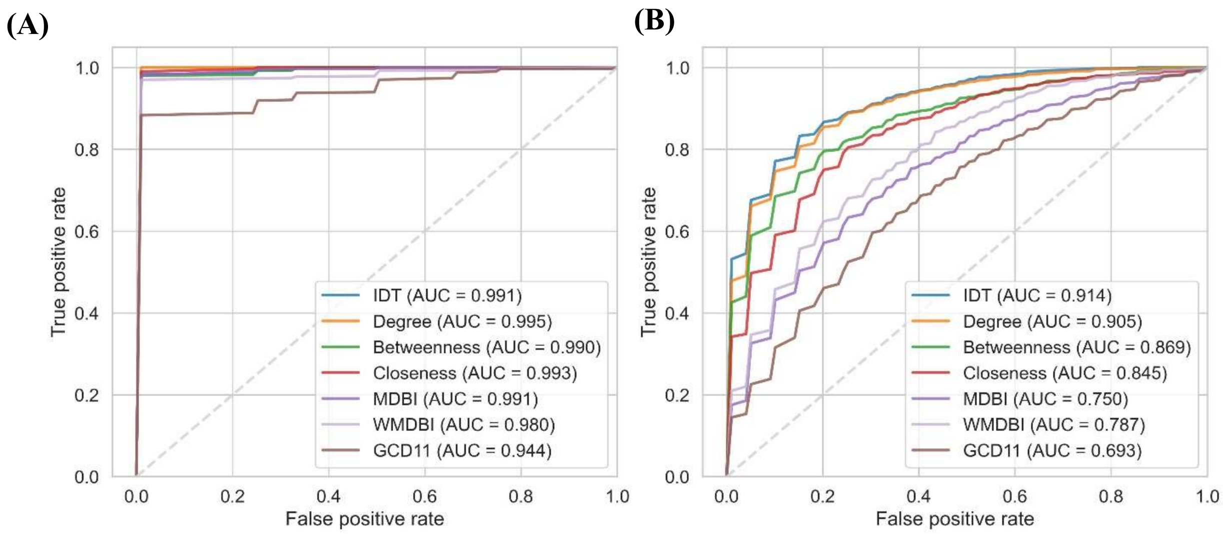

Supplementary Figure 4. Receiver Operating Characteristic curves for Random Forest models fitted to the *GD types* and *HD* datasets. **(A)** *GD types*; **(B)** *HD*. AUC: Area Under the Curve. Intensity-based data pre-treatment (IDT); Degree analysis (Degree), Betweenness Centrality analysis (Betweenness), Closeness centrality analysis (Closeness), MDB Impact analysis (MDBI), Weighted MDB Impact (WMDBI), GCD-11 topology analysis (GCD11). ROC curves were estimated by 3- or 5-fold stratified cross-validation for all datasets.

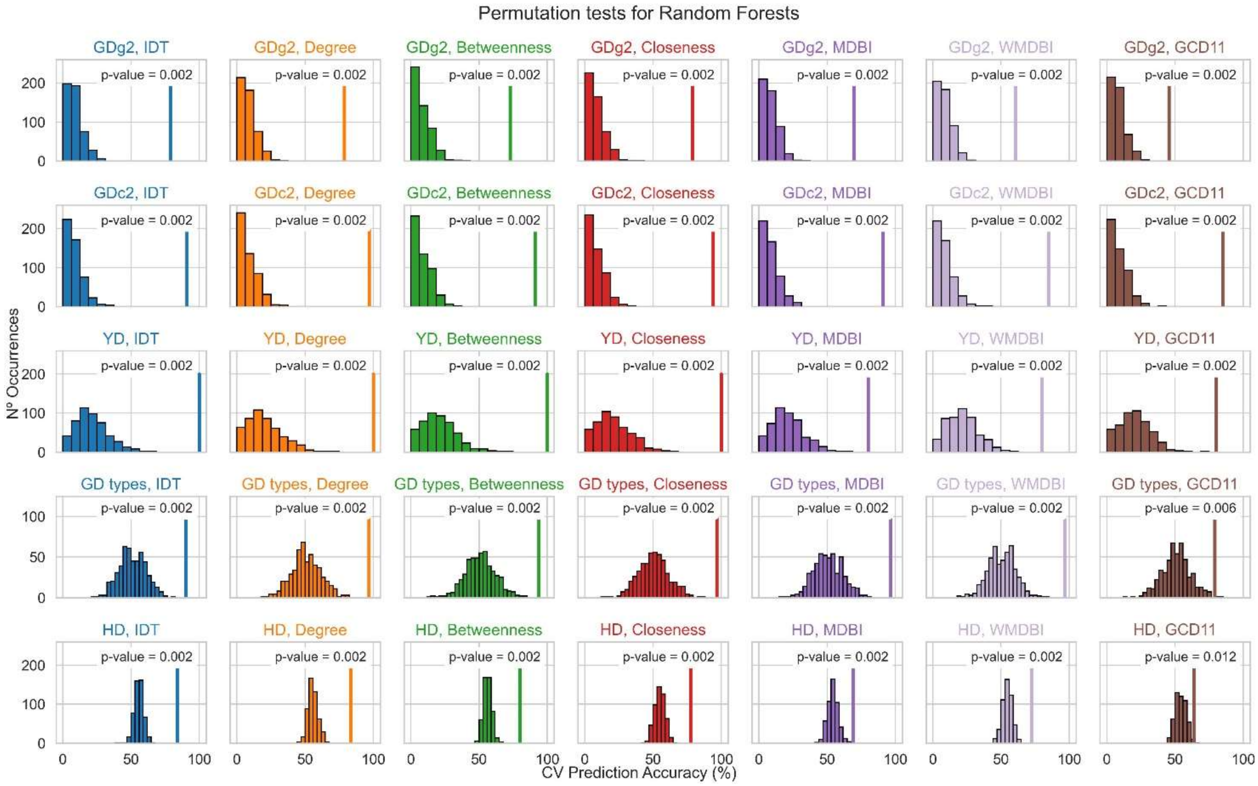

Supplementary Figure 5. Permutation tests for Random Forest models. The distribution of average prediction accuracies of 500 permutations of sample labels is shown. Vertical lines indicate the value of the accuracy of the model without label permutations. Intensity-based data pre-treatment: normalization, *g-log* transformation and Pareto scaling after RF imputation for *HD* and ⅕ min imputation for the other datasets (IDT); Network analysis: Degree analysis (Degree), Betweenness Centrality analysis (Betweenness), Closeness centrality analysis (Closeness), MDB Impact analysis (MDBI), Weighted MDB Impact (WMDBI), GCD-11 topology analysis (GCD11) of sMDiNs.

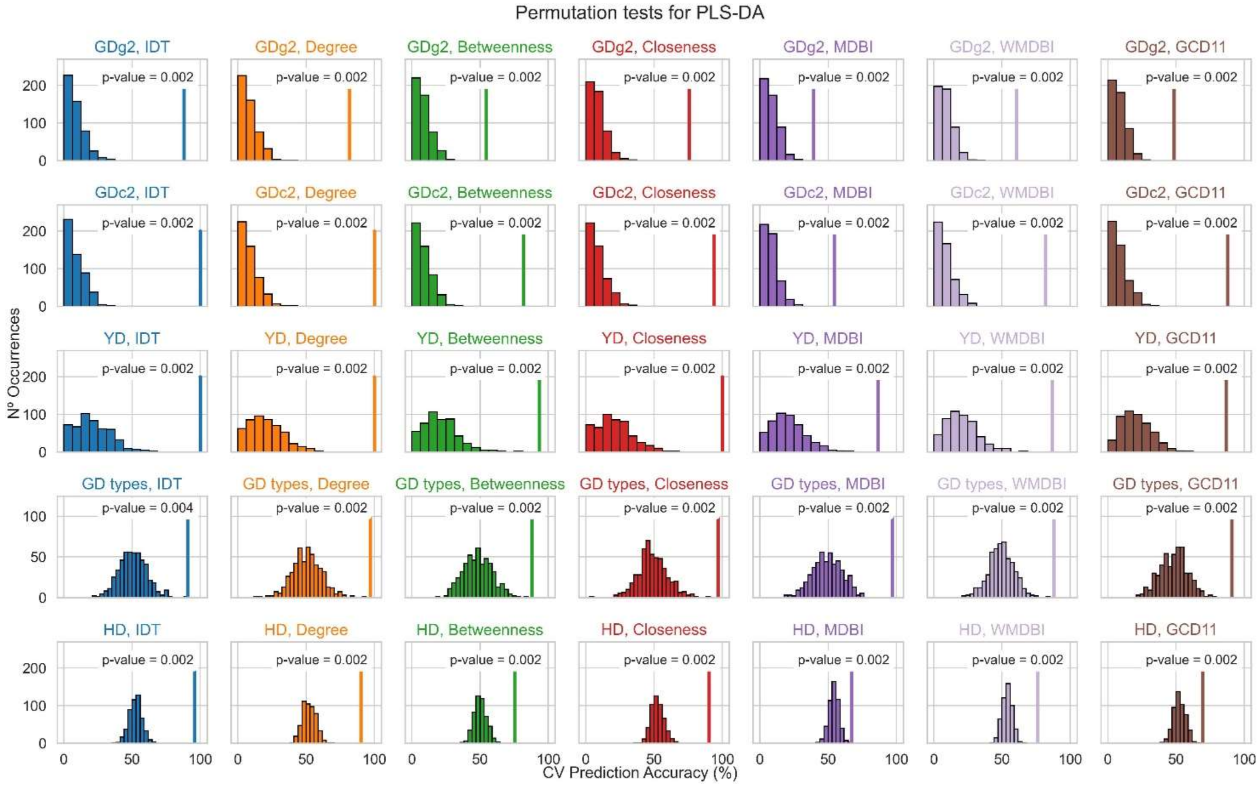

Supplementary Figure 6. Permutation tests for Projection in Latent Structures Discriminant Analysis models. The distribution of average prediction accuracies of 500 permutations of sample labels is shown. Vertical lines indicate the value of the accuracy of the model without label permutations. Intensity-based data pre-treatment: normalization, *g-log* transformation and Pareto scaling after ⅕ min imputation for the other datasets (IDT); Network analysis: Degree analysis (Degree), Betweenness Centrality analysis (Betweenness), Closeness centrality analysis (Closeness), MDB Impact analysis (MDBI), Weighted MDB Impact (WMDBI), GCD-11 topology analysis (GCD11) of sMDiNs.

Supplementary Table 1: Number of nodes established by each MDB in the Mass-Difference Networks (MDiNs) built from the benchmark datasets.

Supplementary Table 2: Permutation tests for the supervised methods. After 500 permutations of sample labels, the *p*-values of prediction accuracy of the unpermuted data are indicated.
